# Space‐Time Coding Conformal Metasurfaces for Multifrequency Beam Steering and Shaping

**DOI:** 10.1002/adma.73837

**Published:** 2026-07-07

**Authors:** Filippo Pepe, Lei Zhang, Yi Ning Zheng, Xiao Qing Chen, Ivan Iudice, Giuseppe Castaldi, Marco Di Renzo, Tie Jun Cui, Vincenzo Galdi

**Affiliations:** ^1^ Fields & Waves Lab Department of Engineering University of Sannio Benevento Italy; ^2^ State Key Laboratory of Millimeter Waves School of Information Science and Engineering Southeast University Nanjing China; ^3^ Security and Cybersecurity in Complex Systems and Air Traffic Management Unit Italian Aerospace Research Centre (CIRA) Capua Italy; ^4^ Institute of Electronics and Digital Technologies (IETR) CNRS and CentraleSupélec Cesson‐Sévigné France; ^5^ Centre for Telecommunications Research Department of Engineering King's College London London United Kingdom

**Keywords:** conformal, metasurface, multifrequency synthesis, space‐time coding

## Abstract

Space‐time coding metasurfaces enable dynamic control of electromagnetic waves by jointly exploiting spatial coding and temporal modulation, unlocking functionalities unattainable with static platforms. While these concepts have been widely explored in planar configurations, their extension to conformal geometries has received comparatively little attention, despite the prevalence of curved platforms in practical applications. Here, space‐time coding conformal metasurfaces are examined as a platform for multifrequency beam steering and shaping on nonplanar structures. A representative cylindrical geometry, characterized by translational invariance of the supporting structure along one direction and mild curvature along the orthogonal transverse coordinate, is adopted as a physically relevant configuration that enables accessible modeling of curvature effects. The proposed design strategy combines semi‐analytical modeling with hybrid synthesis techniques, including evolutionary optimization. The resulting responses are validated through experimental measurements on an X‐band conformal prototype employing a segmented architecture that enables controlled cylindrical bending, showing good agreement with theoretical predictions. These results demonstrate that multifrequency beam steering and shaping can be achieved on curved surfaces, establishing space‐time coding as a viable and scalable approach for dynamic wave manipulation in realistic conformal environments.

## Graphical abstract

Space‐time coding conformal metasurfaces enable dynamic wave control on curved surfaces by combining programmable spatial patterns with temporal modulation. This study shows that a single conformal aperture can independently manipulate multiple frequency components, achieving multifunctional beam shaping and steering, with implications for reconfigurable intelligent surfaces, wireless communications, and emerging smart electromagnetic environments.

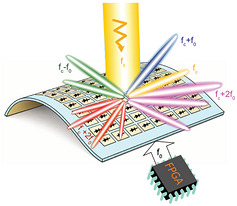

## 1 Introduction

Metasurfaces are engineered electromagnetic (EM) interfaces that enable compact control of wave propagation by tailoring subwavelength boundary responses [1, 2]. Within this broad wavefront‐engineering framework, planar optical and digitally encoded metasurfaces have demonstrated increasingly sophisticated functionalities, including high‐efficiency wide‐angle beam steering, digitally encoded focus control, adaptive aberration correction, and emerging digital‐optics concepts [3, 4, 5, 6]. Initially developed for static wavefront shaping, metasurfaces have evolved toward programmable, reconfigurable architectures in which the response of individual meta‐atoms can be dynamically controlled [7, 8]. In parallel, reconfigurable and intelligent metasurface platforms are being developed for system‐level EM functions beyond wavefront shaping, including wireless power transfer, radio‐frequency (RF) energy harvesting, and communication‐oriented control [9, 10]. Within this broader class, space‐time coding (STC) metasurfaces represent a key conceptual advance. By deliberately modulating the metasurface response in space and time according to prescribed digital sequences, STC metasurfaces extend wave manipulation beyond static boundary conditions and introduce time as an additional design dimension [11].

Temporal modulation enables functionalities fundamentally inaccessible to static metasurfaces, including frequency conversion, harmonic generation, nonreciprocal responses, and multifrequency beam steering and shaping [12, 13, 14], with recent STC metasurface studies further extending these capabilities to dynamic structured‐wave generation, harmonic‐domain field control, and multiplexed vortex‐beam manipulation [15, 16]. Beyond a broad range of wave‐manipulation effects (see, e.g., [11, 17, 18, 19, 20, 21, 22]), in the microwave and millimeter‐wave regimes, these concepts naturally intersect with the paradigm of reconfigurable intelligent surfaces (RISs), where metasurfaces are employed to programmably shape the propagation environment in wireless systems, enabling gains in coverage, capacity, and user scheduling [23, 24, 25]. Within this context, STC metasurfaces are being actively investigated for emerging applications such as advanced multiplexing schemes [26, 27], wave‐based computation [28, 29], integrated sensing and communication (ISAC) architectures [30, 31, 32], physical‐layer security techniques [33, 34, 35, 36], and diagnostics and self‐monitoring of RIS platforms [37, 38]. From a communications perspective, the ability to perform multifrequency beam steering and shaping using a single, programmable surface enables frequency‐multiplexed spatial links, adaptive spectrum utilization, and dynamic control of carrier and harmonic components, which are particularly attractive for platforms subject to strict form‐factor constraints, including aerial vehicles, connected transportation systems, and urban infrastructure.

Despite this rapid progress, most reported STC metasurface implementations assume *planar* geometries. While this assumption is convenient for modeling and prototyping, it is increasingly restrictive for practical deployment, since many relevant platforms, including vehicles, aircraft, unmanned aerial systems, wearable devices, and building or urban infrastructure, are inherently *curved* or require conformal integration. This mismatch has motivated the parallel development of *conformal* metasurfaces for wave manipulation on curved and free‐form structures. Generalized theoretical frameworks have been proposed to model EM boundary conditions on curved interfaces [39], and experimental demonstrations have achieved wavefront shaping and beam steering across microwave, millimeter‐wave, and terahertz regimes [40, 41, 42, 43, 44, 45, 46]. Reconfigurable conformal implementations have also been reported, but they are typically restricted to static or quasi‐static single‐frequency operation [47, 48, 49, 50].

Within wireless communication scenarios, conformal and flexible RIS implementations have further shown that digitally controlled curved surfaces can achieve effective beam steering and coverage shaping when curvature effects are included in the design [51, 52, 53, 54, 55]. These studies also indicate that curvature is not merely a mechanical constraint, but can influence the effective aperture, angular response, and spatial diversity of the surface. However, such implementations remain static or quasi‐static and therefore do not exploit the harmonic‐domain degrees of freedom enabled by STC. A related but distinct line of research has explored time‐modulated conformal metasurfaces, with emphasis on radar‐signature or scattering‐feature manipulation [56, 57]. These works demonstrate the potential of temporal modulation on curved platforms, but they do not provide a systematic framework for independently synthesizing different beam‐steering and beam‐shaping functions at multiple harmonic frequencies.

These considerations motivate the extension of STC metasurfaces beyond the planar paradigm as a step toward realistic multifunctional programmable surfaces. The present work addresses this challenge by combining geometry‐aware conformal modeling with STC‐based multifrequency synthesis. In contrast to planar STC metasurfaces, the proposed formulation explicitly accounts for local incidence‐angle‐dependent reflection states and curvature‐dependent phase factors. In contrast to static conformal RISs and existing time‐modulated conformal scattering platforms, it enables different scattering responses to be synthesized at multiple harmonic frequencies using a single curved aperture.

To demonstrate this concept, we focus on a representative cylindrical configuration, featuring translational invariance of the supporting geometry along one direction and a mild, generic curvature along the orthogonal transverse coordinate, while allowing spatial and temporal coding variations along the surface. This geometry is representative of a broad class of practical scenarios, including vehicular, airborne, and infrastructure‐mounted platforms, and enables a systematic assessment of curvature effects within an accessible, physically transparent modeling framework. The proposed methodology combines a semi‐analytical modeling approach with hybrid synthesis strategies, including evolutionary optimization. Experimental validation is performed using an X‐band conformal prototype with a segmented architecture that enables controlled cylindrical bending, with measured responses showing good agreement with theoretical expectations. Together, these results establish STC conformal metasurfaces as a viable and scalable platform for dynamic wave manipulation on realistic, nonplanar structures.

## 2 Background Theory and Modeling

**FIGURE 1:**
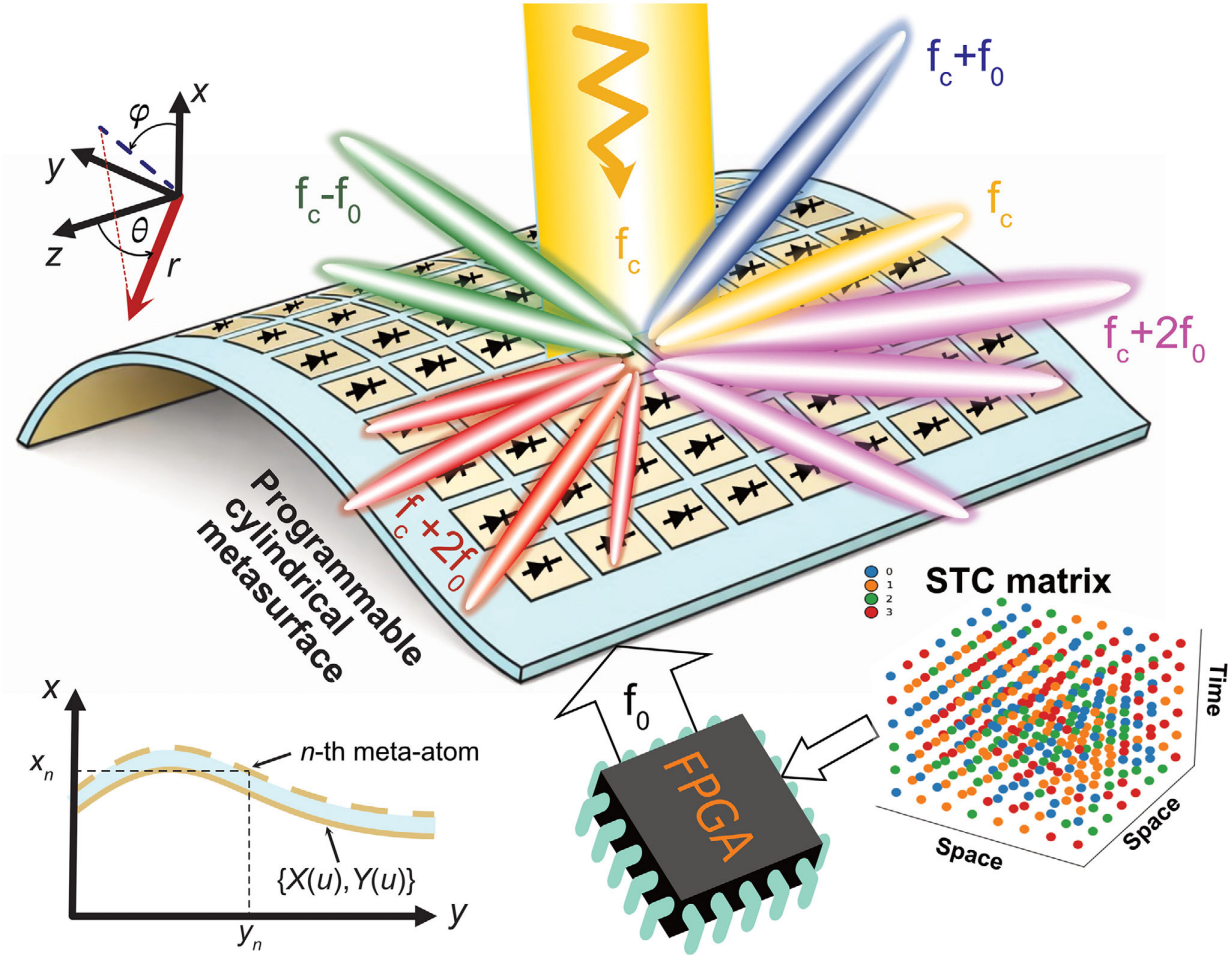
Conceptual illustration of STC conformal metasurface implemented on a cylindrical geometry. A programmable metasurface is illuminated at the carrier frequency *f_c_*, and space‐time modulation driven by an FPGA at frequency *f*_0_ generates multiple harmonic scattering responses at *f_c_* + ν*f*_0_, each with a distinct pattern. The metasurface behavior is encoded through a three‐dimensional STC matrix specifying the discrete reflection states in space and time. Insets show the reference Cartesian and spherical coordinate systems (top left), the cylindrical surface profile (bottom left), and the STC matrix (bottom right) for *b*  =  2.

Referring to the conceptual illustration in Figure 1, a programmable metasurface conformally shaped on a cylindrical surface is considered. The geometry is described in the *x*−*y* plane by a parametric curve {*X*(*u*), *Y*(*u*)} (see the lower‐left inset in Figure 1) and is invariant along the *z*‐direction. The metasurface consists of a dielectric substrate backed by a metallic ground plane and comprises an array of *N*  × *M* subwavelength elements (meta‐atoms). Each meta‐atom embeds *b* electronic switches, such as positive‐intrinsic‐negative (PIN) diodes, which enable discrete control of the reflection phase over *S*  = 2^*b*^  quantized states (e.g., {0, 180°} for *b*  =  1 and {0, 90°, 180°, 270°} for *b*  =  2). The PIN diodes are driven through dedicated biasing lines by a field‐programmable gate array (FPGA), which enables the synthesis of prescribed space‐time distributions of reflection phases across the metasurface. The structure is illuminated by a time‐harmonic, linearly polarized plane wave at the carrier frequency *f_c_*, with a unit‐amplitude electric field polarized along the *z*‐direction and propagating from the negative *x*‐direction. Each (*n*, *m*)‐indexed meta‐atom is assumed to undergo an independent periodic modulation with period *T*_0_ =  1/*f*_0_, such that its reflection coefficient is expressed as

(1) $$\Gamma_{\mathrm{nm}}\;\left(t\right)=\sum_{i=-\infty }^{+\infty }\sum_{\ell =1}^{L}\Gamma_{\mathrm{nm}}^{\left(\ell \right)}\Pi^{\left(\ell \right)}\left(t-iT_{0}\right)\;,\;0<t<T_{0}$$

where Π^(ℓ)^(*t*) denotes a rectangular pulse of duration *T_s_* = *T*_0_ /*L*, equal to unity for (ℓ − 1)*T_s_* < *t* < ℓ*T_s_* and zero otherwise. The coefficients $\Gamma_{nm}^{\left(\ell \right)}$ represent the reflection states at the carrier frequency *f_c_* and belong to the discrete set

(2) $$\Gamma_{\mathrm{nm}}^{\left(\ell \right)}\in \Xi_{n}\equiv {\left\{\gamma_{n,s}\right\}}_{s=1}^{S}$$

Unlike planar metasurface configurations, where an identical set of quantized reflection states is typically assumed for all meta‐atoms, the conformal geometry considered here leads to spatially varying incidence conditions across the surface. As a result, the admissible reflection states generally depend on the position along the curved profile and are therefore indexed by *n*. Consistent with the static conformal analysis reported in [50], the values γ_*n*,*s*_ are obtained through full‐wave numerical simulations of the meta‐atom under different incidence angles and PIN‐diode configurations (see the Experimental Section for details). This procedure also accounts for nonideal effects, including losses, which lead to reflection coefficients with nonunit amplitude. Overall, the modulation process is described by a three‐dimensional (3D) STC matrix whose entries specify the logical states corresponding to the discrete reflection responses (for example, {0, 1, 2, 3} in the 2‐bit case, schematically illustrated in Figure 1).

As detailed in the Supporting Information, under the assumptions of an electrically large metasurface with mild curvature, and a modulation frequency much smaller than the carrier frequency (*f*_0_ ≪ *f_c_*), the scattered electric far field can be approximated within an adiabatic physical‐optics framework as

(3) $$E_{\infty }^{s}\left(\theta ,\varphi ,t\right)\approx \sum_{n=1}^{N}\sum_{m=1}^{M}\Gamma_{\mathrm{nm}}\left(t\right)\chi_{n}\left(\theta ,\varphi \right)\exp\left\{j\left[2\pi f_{c}t+k\left(\theta ,\varphi \right)\cdot r_{\mathrm{nm}}\right]\right\}$$

where *θ* and *φ* denote the conventional polar and azimuth angles, respectively, in the associated spherical coordinate system (see the upper‐left inset in Figure 1). The vector $r_{\mathrm{nm}}=x_{n}\hat{x}+y_{n}\hat{y}+z_{m}\hat{z}$ represents the position of the generic (*n*, *m*)‐indexed meta‐atom, with *x_n_* =  *X*(*u_n_*) and *y_n_* =  *Y*(*u_n_*), while $k\;\left(\theta ,\varphi \right)=k_{c}\;\left(\sin\theta \cos\varphi \hat{x}+\sin\theta \sin\varphi \hat{y}+\cos\theta \hat{z}\right)$ denotes the observation wavevector. Here, *k_c_* =  2π*f_c_*/*c*  =  2π/λ_*c*_ is the free‐space wavenumber at the carrier frequency *f_c_*, with *c* and λ_*c*_ denoting the corresponding wave speed and wavelength, respectively. Throughout this work, vectors are indicated by boldface symbols, and $\hat{\alpha }\;$ denotes a unit vector directed along the α‐direction. Moreover, the vector quantity

(4) $$\begin{array}{ccc} & & \chi_{n}\;\left(\theta ,\varphi \right)=\exp\left(jk_{c}x_{n}\right)\;\;\times \\ & & \left[\left({\dot{x}}_{n}\sin\varphi -{\dot{y}}_{n}\cos\varphi \right)\hat{\theta }+\cos\theta \left({\dot{y}}_{n}\sin\varphi +{\dot{x}}_{n}\cos\varphi \right)\hat{\varphi }\right]\; \end{array}$$

accounts for the phase of the impinging wave and for directional factors characterizing the response of the *n*‐th meta‐atom. In this expression, ${\dot{x}}_{n}=\dot{X}\;\left(u_{n}\right)$ and ${\dot{y}}_{n}=\dot{Y}\;\left(u_{n}\right)$, with the overdot indicating differentiation with respect to *u*, and $\hat{\theta }$ and $\hat{\varphi }\;$ denote the conventional spherical unit vectors. The meta‐atoms are uniformly distributed along the conformal profile, with their centers separated by a distance *d_s_* measured along the arc‐length coordinate. Along the axial direction, a uniform spacing *d_z_* is assumed, so that the element positions are given by *z_m_* =  (*m* − 1/2 − *M*/2)*d_z_*, with *m*  =  1, …, *M*. Following the modeling approach commonly adopted for planar configurations [11], substitution of Equation (1) into Equation (3), together with a Fourier‐series expansion, allows the scattered far field to be conveniently expressed as

(5) $$E_{\infty }^{s}\;\left(\theta ,\varphi ,t\right)=\sum_{\nu =-\infty }^{\infty }F^{\left(\nu \right)}\left(\theta ,\varphi \right)\exp\left[j2\pi \left(f_{c}+\nu f0\right)t\right]\;$$

which represents a harmonic decomposition wherein each term corresponds to a distinct scattering response at frequency *f_c_* + ν*f*_0_, viz.,

(6) $$F^{\left(\nu \right)}\;\left(\theta ,\varphi \right)=\sum_{n=1}^{N}\sum_{m=1}^{M}a_{\mathrm{nm}}^{\left(\nu \right)}\;\chi_{n}\left(\theta ,\varphi \right)\;\exp\left[jk\left(\theta ,\varphi \right)\cdot r_{\mathrm{nm}}\right]$$

Here, $a_{nm}^{\left(\nu \right)}$ are complex‐valued equivalent amplitudes associated with the (*n*, *m*)‐indexed meta‐atom and are obtained as [11]

(7) $$a_{\mathrm{nm}}^{\left(\nu \right)}=\sum_{\ell =1}^{L}\frac{\Gamma_{\mathrm{nm}}^{\left(\ell \right)}}{L}\mathrm{sinc}\left(\frac{\pi \nu }{L}\right)\exp\left[\frac{-j\pi \nu \left(2\ell -1\right)}{L}\right]\;$$

where sinc (*x*) = (sin*x*) /*x*. In summary, as schematically illustrated in Figure 1, STC modulation generates distinct scattering patterns at the different harmonic frequencies, which can, in principle, be independently controlled and exploited for multiplexing applications. However, as also observed in planar configurations [19], the multifrequency synthesis problem is difficult to address analytically. This difficulty arises because the equivalent amplitudes $a_{nm}^{\left(\nu \right)}$ in Equation (7) depend on the entire set of reflection coefficients $\Gamma_{nm}^{\left(\ell \right)}$ defining the temporal coding sequences, thereby coupling the synthesis of all harmonic frequencies. From a mathematical viewpoint, multifrequency scattering therefore results in a strongly coupled optimization problem, in which the scattering responses at different harmonics cannot be treated independently. By defining a set ϒ of harmonic orders of interest and the corresponding scattering power patterns Φ^(ν)^ (θ,φ) = | ***F*** ^(ν)^(θ,φ)|^2^ , the multifrequency synthesis problem can be formulated as the following optimization problem:

(8) $$\Gamma_{nm,opt}^{\left(\ell \right)}=\begin{array}{c} \underset{\Gamma_{nm}^{\left(\ell \right)}}{\mathrm{argmin}}\sum_{\nu \in \Upsilon }w_{\nu }\left\|\Phi^{\left(\nu \right)}\left(\theta ,\varphi \right)-\Phi_{t}^{\left(\nu \right)}\left(\theta ,\varphi \right)\right\|,\\ s.t.\;\;\Gamma_{nm}^{\left(\ell \right)}\in \Xi_{n},\;\forall n,m,\ell \end{array}$$

where the subscript “*t*” denotes the target scattering patterns, *w*_ν_ are weighting coefficients used to prioritize different harmonic orders, and ‖ · ‖ represents a suitable norm. This formulation leads to a mixed‐integer fractional quadratic programming problem [58], which is inherently challenging to solve efficiently due to its nonconvex and combinatorial nature over a search space of dimension *S* ^*N* × *M* × *L*^. Even for moderately sized metasurfaces and relatively short coding sequences, exhaustive search strategies rapidly become computationally prohibitive. Consequently, alternative solution strategies, including machine‐learning‐based methods [59, 60] and quantum‐annealing approaches [61], have recently been investigated to obtain suboptimal solutions.

Here, a different hybrid strategy is adopted by extending the semi‐analytical approach introduced in [19] for planar STC metasurfaces and combining it with evolutionary optimization techniques [62]. The method in [19] aims to decouple the multifrequency synthesis problem through a suitable temporal intertwining of *Q* coding subsequences, each of length *P*, such that the overall sequence length satisfies *L*  =  *PQ*. By enforcing appropriate phase‐shift relations among these subsequences, each subsequence can be made to control a single harmonic order. In particular, for *S*  = 2^*b*^  discrete reflection states and by choosing *P*  =  *S*, it is possible to independently control *Q* harmonic orders [11]. As detailed in the Supporting Information, this strategy can be approximately extended to the conformal scenario considered here, provided that the meta‐atom satisfies an angular‐stability condition in its reflection response. This condition requires that, as the angle of incidence varies across the curved surface, the phase responses associated with the discretized reflection states may change, while their mutual phase differences remain approximately constant. Under this condition, the scattering responses at the harmonic orders ν  =  0, 1, …, *Q* − 1 can be approximated as

(9) $$F^{\left(\nu \right)}\left(\theta ,\varphi \right)\approx \xi^{\left(\nu \right)}\sum_{n=1}^{N}\sum_{m=1}^{M}\Gamma_{\mathrm{nm}}^{\left(\nu +1\right)}\chi_{n}\left(\theta ,\varphi \right)\;\exp\left[jk\left(\theta ,\varphi \right)\cdot r_{\mathrm{nm}}\right]$$

where

(10) $$\xi^{\left(\nu \right)}=\frac{1}{P}\;\mathrm{sinc}\left(\frac{\pi \nu }{L}\right)\exp\left[\frac{-j\pi \nu \left(2\nu +1\right)}{L}\right]$$

accounts for the spectral decay of the harmonic components. As can be inferred from Equation (9), the scattering response at the ν‐th harmonic order depends only on the subset of reflection coefficients $\Gamma_{nm}^{\left(\nu +1\right)}$ associated with the corresponding temporal subsequence, thereby enabling independent synthesis of each harmonic. The accuracy of this approximate decoupling depends on the angular stability of the local reflection‐state alphabet, which is quantified in Section 3.1. A further limitation is the appearance of replicas of the synthesized scattering patterns at higher and negative harmonic orders; their amplitudes follow the sinc‐type envelope in Equation (10) and can be reduced through more elaborate temporal coding schemes [19], as discussed further in Section 3.2.

To further reduce the complexity of the synthesis problem, a factorization of the reflection coefficient is introduced to decouple the synthesis along the cylindrical section (*x* − *y* plane) from that along the axial (*z*) direction, namely,

(11) $$\Gamma_{\mathrm{nm}}^{\left(\nu +1\right)}\approx {\tilde{\Gamma }}_{n}^{\left(\nu +1\right)}{\hat{\Gamma }}_{m}^{\left(\nu +1\right)}$$

It is worth noting that, even when the coefficients ${\tilde{\Gamma }}_{n}^{\left(\nu +1\right)}$ and ${\hat{\Gamma }}_{m}^{\left(\nu +1\right)}$ are constrained to discrete values, this factorization cannot, in general, be exact. This limitation stems from the spatial variation of the admissible reflection‐coefficient sets along the cylindrical profile, as defined in Equation (2). Nevertheless, the approximation can be sufficiently accurate provided that the meta‐atom satisfies the angular‐stability condition discussed earlier. By substituting Equation (11) into Equation (9), the scattering responses naturally factorize as

(12) $$F^{\left(\nu \right)}\left(\theta ,\varphi \right)\approx \xi^{\left(\nu \right)}V^{\left(\nu \right)}\left(\theta ,\varphi \right)W^{\left(\nu \right)}\left(\theta \right)$$

where

(13) $$V^{\left(\nu \right)}\;\left(\theta ,\varphi \right)=\sum_{n=1}^{N}{\tilde{\Gamma }}_{n}^{\left(\nu +1\right)}\chi_{n}\left(\theta ,\varphi \right)\;\exp\left[jk_{c}\sin\theta \left(x_{n}\cos\varphi +y_{n}\sin\varphi \right)\right]\;$$

and

(14) $$W^{\left(\nu \right)}\;\left(\theta \right)=\sum_{m=1}^{M}{\hat{\Gamma }}_{m}^{\left(\nu +1\right)}\;\exp\left(jk_{c}\cos\theta z_{m}\right)\;$$

This formulation leads to two decoupled synthesis problems, which can be addressed independently for each harmonic order ν  =  0, 1, …, *Q* − 1. By fixing the elevation angle to a reference value *θ*_0_ (corresponding, e.g., to the main‐beam direction), the synthesis problem in Equation (13) reduces to that of a static conformal cylindrical metasurface invariant along the axial direction. This problem is conceptually analogous to that investigated in Ref. [50] and is therefore tackled using a similar evolutionary optimization strategy, subject to the constraint ${\tilde{\Gamma }}_{n}^{\left(\nu +1\right)}\in \Xi_{n}$, as defined in Equation (2). This choice is practical rather than fundamental: other discrete or relaxed optimizers could be used within the same reduced single‐harmonic synthesis framework. Here, evolutionary optimization is adopted as a robust solver that provides reproducible suboptimal solutions with acceptable computational cost for the examples considered.

Conversely, the synthesis problem in Equation (14) is equivalent to that of a conventional planar metasurface composed of uniformly spaced elements along the *z*‐direction and invariant in the *x*−*y* plane. For canonical scattering functionalities, such as anomalous reflection, beam splitting, and diffuse (low‐crest) scattering, this problem admits analytical solutions.

Once the optimized coefficients ${\tilde{\Gamma }}_{n,opt}^{\left(\nu +1\right)}$ and ${\hat{\Gamma }}_{m,\mathrm{opt}}^{\left(\nu +1\right)}$ are obtained for a given harmonic order ν, the reflection coefficients to be implemented in the STC sequence are derived from Equation (11) as

(15) $$\Gamma_{\mathrm{nm},\mathrm{opt}}^{\left(\nu +1\right)}=P_{\Xi_{n}}\;\left[{\tilde{\Gamma }}_{n,\mathrm{opt}}^{\left(\nu +1\right)}{\hat{\Gamma }}_{m,\mathrm{opt}}^{\left(\nu +1\right)}\right]$$

where the projection (quantization) operator

(16) $$P_{\Xi }\;\left[\psi \right]=\underset{\zeta \in \Xi }{\mathrm{argmin}}\left|\psi -\zeta \right|\;$$

ensures physical realizability of the reflection states.

The practical impact of the approximate factorization and subsequent projection in Equations (11) and (15) is assessed in the Supporting Information through a representative comparison between the ideal factorized pattern and the corresponding implementable projected pattern (Figure S1). The comparison shows that, for the mildly curved geometries and angularly stable 2‐bit meta‐atom considered here, the prescribed main‐beam directions and overall pattern morphology are preserved, while the main discrepancies appear in the sidelobe regions and fine pattern details. This behavior is consistent with the local quantization introduced by the projection operator in Equation (16).

Although the simplifying assumptions underlying this approach preclude the synthesis of arbitrary scattering responses, they enable the efficient and sufficiently accurate realization of canonical functionalities, such as anomalous reflection and beam splitting, with significantly reduced computational complexity. More specifically, after the multifrequency problem has been decoupled into independent harmonic syntheses, a direct non‐factorized 2D optimization over a fully addressable *N* × *M* aperture would still involve a search space of size *S^NM^* per harmonic. The factorized approach avoids this scaling for the canonical responses considered here by reducing the synthesis to separate conformal‐direction and axial‐direction subproblems. Fully programmable 2D implementations targeting arbitrary nonseparable patterns would therefore require more advanced optimization strategies, such as hierarchical design, convex relaxations followed by quantization, or machine‐learning‐assisted inverse design.

## 3 Results and Discussion

### 3.1 Geometry and Meta‐Atom

In what follows, we consider three representative cylindrical conformal profiles. These profiles are constructed from circular arcs and are described in the *x*−*y* plane by parametric curves, whose explicit expressions are provided in the Supporting Information.

**FIGURE 2:**
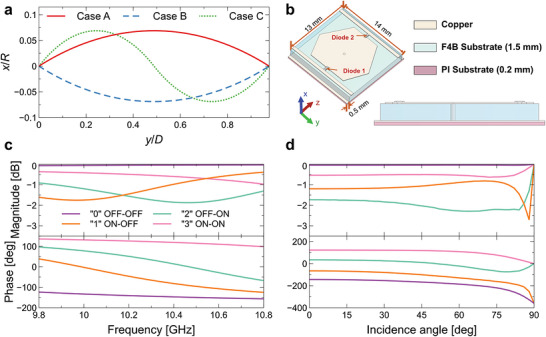
Geometry and meta‐atom response. (a) Representative cylindrical conformal profile considered in this study (see Supporting Information for the parametric equations), with *D*/*R*  =  0.747 for Cases *A* and *B*, and *D*/*R*  =  1.493 for Case *C*. (b) Schematic of 2‐bit meta‐atom. (c) Simulated frequency response of meta‐atom (reflection‐coefficient magnitude and phase) under normal incidence. (d) Simulated angular response of meta‐atom (reflection‐coefficient magnitude and phase) at 10.3 GHz.

As shown in Figure 2a, cases *A* and *B* describe convex and concave cylindrical geometries, respectively, whereas case *C* corresponds to a piecewise configuration obtained by concatenating two circular arcs of equal radius and opposite convexity, resulting in an extended conformal aperture. In all cases, the parameter *R* controls the local radius of curvature, with the planar limit recovered as *R*  → ∞, while *D* determines the total transverse extent and the corresponding angular span of the conformal surface.

Figure 2b shows a schematic of the 2‐bit meta‐atom considered in this study. The unit cell has lateral dimensions of 14 × 14 mm^2^ and consists of a hexagonal metallic patch connected to two metallic strips through two PIN diodes. A copper‐clad F4B dielectric substrate is assumed, with nominal relative permittivity ε_*r*_ =  2.6 and loss tangent tan δ =  0.001. Figure 2c reports the simulated reflection response of the meta‐atom, in terms of magnitude and phase, for the four possible combinations of the PIN‐diode states. The simulations are carried out using a commercial finite‐element‐based EM solver (see the Experimental Section for details). At the design frequency of 10.3 GHz, the four states exhibit phase differences close to 90° with reflection amplitudes close to unity. The simulations are performed assuming a slightly reduced substrate relative permittivity ( ε_*r*_ =  2.4), which provides improved agreement with experimental measurements, as discussed in the Supporting Information (see Figure S1). The frequency response in Figure 2c also indicates that the approximate 2‐bit behavior is maintained only over a limited carrier‐frequency range. Away from 10.3 GHz, deviations in the relative phase spacing and reflection amplitudes would progressively reduce the accuracy of the synthesized STC patterns. Accordingly, the prototype should be regarded as a narrowband proof‐of‐concept around the carrier frequency; broadband operation would require a meta‐atom specifically optimized for frequency‐ and angle‐stable phase differences.

Figure 2d shows the simulated angular response of the meta‐atom at 10.3 GHz. Over a broad angular range, the relative phase differences between the reflection states remain approximately constant, supporting the angular‐stability assumption adopted in the synthesis procedure. To quantify this behavior, we define the root‐mean‐square (RMS) relative phase error

(17) $$\Delta \phi_{\mathrm{rms}}\;\left(\alpha \right)=\sqrt{\frac{1}{3}\sum_{s=1}^{3}{\left(\mathrm{wrap}\left[\phi_{s}\left(\alpha \right)-\phi_{0}\left(\alpha \right)-s\frac{\pi }{2}\right]\right)}^{2}}\;$$

where *ϕ*_*s*_(α) is the reflection phase of the *s*‐th coding state at local incidence angle α, and wrap[·] maps the phase error onto (− π, π]. This metric measures the deviation of the local 2‐bit phase alphabet from the ideal quadrature spacing. From the full‐wave data in Figure 2d, we obtain *Δ* *ϕ*_rms_ ≈ 7.5°, 10°, and 25° at local incidence angles of 15°, 30°, and 60°, respectively, and *Δ* *ϕ*_rms_ ≈ 40° near 85°. As expected, the angular‐stability assumption therefore gradually deteriorates near grazing incidence, where the reflection response becomes weakly dependent on the PIN‐diode configuration. Consistent with observations in static conformal implementations [50], this leads to reduced effectiveness of meta‐atoms located near the edges of the illuminated region, thereby limiting the number of available degrees of control. In the resulting beamforming response, this mainly reduces aperture efficiency and increases sidelobe or ringing levels, rather than producing a deterministic shift of the prescribed main‐beam direction. The results in Figure 2d are used to define the sets Ξ_*n*_ of admissible discretized reflection coefficients in Equation (2), with the position of each meta‐atom determining the local incidence direction; hence, the reduced controllability of large‐incidence‐angle elements is directly incorporated in the synthesis. For a cylindrical aperture, reducing the radius *R* increases the range of local incidence angles sampled across the metasurface, so that the minimum usable radius is determined not only by fabrication constraints, but also by the angular‐stability range of the meta‐atom. Moreover, if *R* becomes too small, the local‐planar physical‐optics approximation underlying the semi‐analytical model may also break down due to nonlocal coupling, shadowing, multiple scattering, and edge diffraction. The experimental profiles considered in this work are therefore chosen to remain in a mildly curved regime for which the above assumptions are expected to be adequate ($\Delta \phi_{\mathrm{rms}}\underset{∼}{<}8^{\circ }$); their validity is assessed experimentally in Section 3.3.

### 3.2 Representative Numerical Syntheses

As a first synthesis example, we consider a convex circular cylinder (Case *A* in Figure 2a) with radius *R*  =  134 mm and transverse extent *D*  =  268 mm, corresponding to a semicircumference. Along the axial direction, a length *D_z_* =  448 mm is assumed. The meta‐atoms are spaced by *d_s_* = *d_z_*  =  14 mm, resulting in *N*  =  30 elements along the conformal direction and *M*  =  32 elements along the axial direction.

**FIGURE 3:**
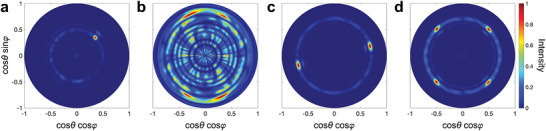
Simulated harmonic scattering patterns, in the *x* > 0 halfspace, for a convex cylindrical configuration (Case *A* in Figure 2a) with *R*  =  134 mm, *D*  =  268 mm, *D_z_* =  448 mm, *d_s_* = *d_z_*  =  14 mm, *N*  =  30 and *M*  =  32, assuming *f_c_* =  10.3GHz and *P*  =  *Q*  =  *S*  =  4, i.e., *L*  =  *PQ*  =  16. False‐color maps of the synthesized scattering patterns (field intensity) at the harmonic orders: (a) ν  =  0, anomalous reflection toward (*θ*_0_,*φ*_0_) = (60°, 45°); (b) ν  =  1, diffuse scattering; (c) ν  =  2, beam splitting toward (*θ*_0_,*φ*_0_) = (45°, 15°)  and (135°, 15°); and (d) ν  =  3, quad‐beam response with lobes directed toward (*θ*_0_,*φ*_0_) = (45°, ±45°)  and (135°, ±45°). All patterns are normalized with respect to their maximum value.

Figure 3 illustrates the multifrequency synthesis results obtained using the hybrid approach outlined in Section 2, assuming *f_c_* =  10.3 GHz and *P*  =  *Q*  =  *S*  =  4. This choice yields temporal coding sequences of length *L*  =  *PQ*  =  16 and enables independent control of the harmonic orders ν  =  0,  1,  2,  3. At the fundamental harmonic ν  =  0 (Figure 3a), we synthesize an anomalous reflection toward the direction (*θ*_0_,*φ*_0_) = (60°, 45°) . The synthesis proceeds by first designing the pattern in Equation (13), for fixed *θ*  = *θ*_0_, via evolutionary optimization (see the Experimental Section for details) to obtain the desired azimuthal deflection at *φ*_0_. The axial contribution in Equation (14) is then synthesized analytically by imposing a linear phase gradient,

(18) $${\hat{\Gamma }}_{m}^{\left(\nu +1\right)}=\exp\left(-jk_{c}z_{m}\cos\theta_{0}\right)$$

after which the final quantization step in Equation (15) is applied. In Figure 3a, and in the following figures, the 2D scattering patterns are displayed as false‐color maps in terms of cosine directors. Since the analysis is restricted to scattering in the *x* > 0 half‐space, the cosine‐director definition differs from the conventional one.

At the first harmonic order ν  =  1 (Figure 3b), we synthesize a diffuse‐scattering pattern, which is of potential interest for low‐observability applications [63]. In this case, the azimuthal synthesis is carried out by minimizing the crest factor via evolutionary optimization (see the Experimental Section) for fixed *θ*  = *θ*_0_  =  90°, while the axial synthesis is based on Golay‐Rudin‐Shapiro (GRS) polynomials [63, 64, 65] (see Supporting Information for additional details).

At the second harmonic order ν  =  2 (Figure 3c), we synthesize a beam‐splitting response with two lobes directed toward (*θ*_0_,*φ*_0_) = (45°, 15°)  and (135°, 15°). In this case, the azimuthal synthesis follows the same procedure adopted for the anomalous‐reflection response in Figure 3a, albeit with different target angles *θ*_0_ and *φ*_0_. Along the axial direction, the beam‐splitting behavior is obtained by imposing a conventional binary phase profile [66],

(19) $${\hat{\Gamma }}_{m}^{\left(\nu +1\right)}=\mathrm{sign}\left[\cos\left(k_{c}z_{m}\cos\theta_{0}\right)\right]$$

Finally, at the third harmonic order ν  =  3 (Figure 3d), we synthesize a quad‐beam response, with four lobes directed toward (*θ*_0_,*φ*_0_) = (45°, ±45°)  and (135°, ±45°). The azimuthal synthesis is performed at fixed *θ*  = *θ*_0_  =  45° by designing, via evolutionary optimization, a beam‐splitting pattern at *φ*_0_ =   ± 45°. A further splitting along the axial direction is then enforced by applying the binary phase profile in Equation (19).

Here and in the following, the harmonic scattering patterns are normalized with respect to their maximum value. The corresponding relative amplitudes are governed by the spectral‐decay factor in Equation (10), which, for the assumed sequence length *L*  =  16, yields amplitude differences $\underset{∼}{<}0.5\mathrm{dB}$ across the harmonic orders of interest. As previously noted, and further detailed in the Supporting Information, the frequency‐decoupling procedure inherently produces replicas of the synthesized patterns at higher and negative harmonic orders. Owing to the periodic structure of the finite interleaved coding sequence, the pattern synthesized at harmonic order ν is replicated at orders ν + *kQ*, with integer *k*, while its amplitude is weighted by the sinc‐type spectral envelope in Equation (10). Thus, for *P*  =  *Q*  =  *S*  =  4 and *L*  =  16, the first replicas of the controlled harmonics ν  =  0, 1, 2, 3 occur at ν  =  4, 5, 6, 7, respectively. These components are spectrally separated from the desired harmonics by integer multiples of the modulation frequency *f*_0_, and can therefore be mitigated by sufficiently narrowband receivers or appropriate RF/baseband filtering. In systems with wider receive bandwidths or multiple channels, they should instead be treated as out‐of‐band or adjacent‐harmonic leakage, with the required suppression level determined by the waveform, link budget, receiver bandwidth, channel allocation, and filtering architecture. The present *L*  =  16 coding sequence is intended as a compact proof‐of‐concept rather than an optimized spectral‐suppression design. Previous planar STC studies show that temporal coding can substantially improve spectral selectivity: low‐pass finite‐impulse‐response (FIR) filtering increased the average power fraction delivered to four controlled harmonics from about 24% to about 54% [19], while machine‐learning‐enabled STC synthesis, using *L*  =  24 coding sequences, concentrated up to 74% of the power within a *Q*  =  5 target harmonic band and yielded higher‐order harmonic levels below approximately −8 dB [60]. For communication‐ and RIS‐oriented implementations, the temporal code should therefore be co‐designed with the receiver bandwidth, channel allocation, and filtering architecture, using FIR‐filtered or machine‐learning‐assisted strategies when stronger replica suppression is required.

**FIGURE 4:**
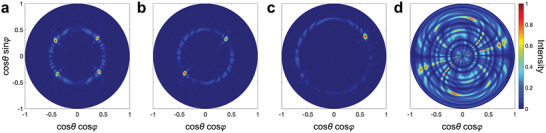
Simulated harmonic scattering patterns, in the *x* > 0 halfspace, for a convex‐concave cylindrical configuration (Case *C* in Figure 2a) with *R*  =  260 mm, *D*  =  422 mm, *D_z_* =  448 mm, *d_s_* = *d_z_*  =  14 mm, *N*  =  30 and *M*  =  32, assuming *f_c_* =  10.3 GHz and *P*  =  *Q*  =  *S*  =  4, i.e., *L*  =  *PQ*  =  16. False‐color maps of the synthesized scattering patterns (field intensity) at the harmonic orders: (a) ν  =  0, quad‐beam response with lobes directed toward (*θ*_0_,*φ*_0_) = (60°, −35°) ,  (60°, 40°),  (120°, −35°) and (120°, 40°); (b) ν  =  1, beam splitting toward (*θ*_0_,*φ*_0_) = (60°, 40°)  and (120°, 40°); (c) ν  =  2, anomalous reflection toward (*θ*_0_,*φ*_0_) = (45°, 30°) ; and (d) ν  =  3, diffuse scattering. All patterns are normalized with respect to their maximum value.

Figure 4 illustrates an additional example, concerning a convex‐concave conformal profile (Case *C* in Figure 2a) with *R*  =  260 mm and *D*  =  422 mm, while all other parameters are identical to those of the previous configuration. In this case, we synthesize variants of the scattering patterns shown in Figure 3, redistributed among the different harmonic orders.

The examples in Figure 3 and Figure 4 are intended as representative proof‐of‐concept syntheses, aimed at illustrating the flexibility of the proposed framework rather than optimizing a specific system‐level performance metric. As a qualitative comparison with the corresponding ideal continuous planar aperture having the same rectangular footprint, the synthesized single‐ and multi‐beam patterns exhibit, on average, beam‐pointing errors below approximately 2°, beamwidths agreeing within ∼ 0.1, and sidelobe levels ranging from ∼ −10 dB to ∼ −5 dB. For the diffuse‐scattering examples, where no prescribed main‐beam region is defined, the scattering uniformity is characterized through the crest factor, which is equal to 3.1 and 2.4 for the two considered cases. These results should therefore be regarded as explanatory demonstrations of the proposed design procedure; further optimization of the coding strategy could improve the reported figures of merit.

Overall, these results demonstrate that STC conformal metasurfaces provide a versatile platform for implementing application‐relevant functionalities, including multifrequency beam steering, beam splitting, and diffuse scattering, within a single conformal aperture.

For clarity, the complete STC sequences for the above numerical examples, represented as 3D matrices of size 32 × 30 × 16, are not shown graphically in the main text. Instead, the corresponding coding matrices are provided as ASCII files in the Supporting Information to ensure reproducibility. The Supporting Information also includes an algorithmic summary of the general STC matrix generation procedure used throughout this work, including the frequency decoupling, spatial synthesis, projection, and temporal interleaving steps.

### 3.3 Experimental Validation

**FIGURE 5:**
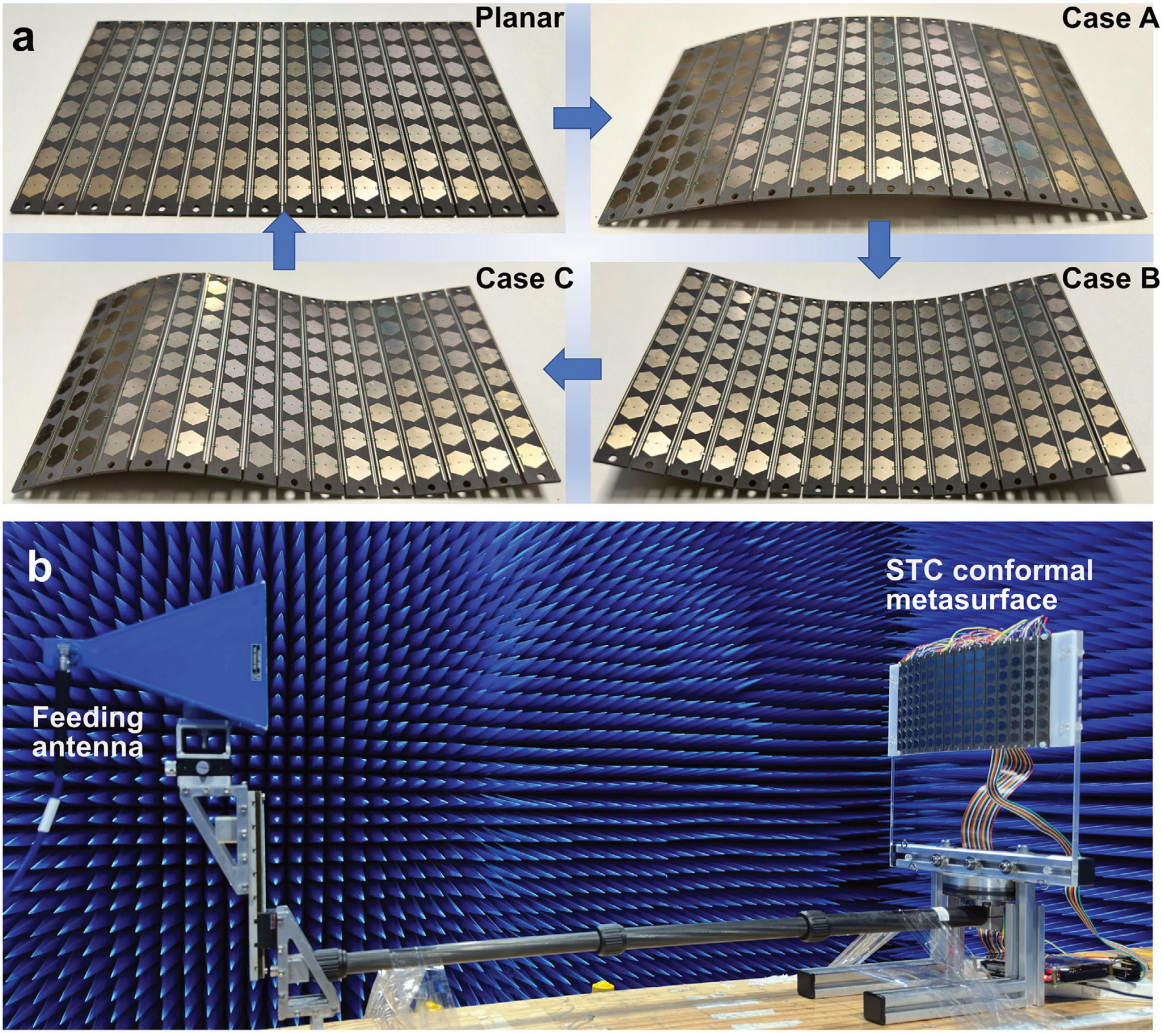
Conformal metasurface prototypes and experimental setup. (a) Renderings of fabricated metasurface prototype in the planar configuration and in the three cylindrical conformal configurations considered in this study (Cases *A*‐*C* in Figure 2a), obtained by mechanically bending the same segmented metasurface to different prescribed curvature profiles. (b) Photograph of the experimental setup inside the anechoic chamber, showing the feeding antenna and the metasurface prototype mounted on a conformal mechanical support.

For experimental validation, we employ an STC metasurface prototype operating in the X‐band. As detailed in the Experimental Section, the metasurface comprises a planar array of 2‐bit meta‐atoms (Figure 2b) mounted on a mechanically reconfigurable support consisting of *N*  =  16 rigid segments connected by precision hinges. Each segment hosts *M*  =  8 meta‐atoms sharing common biasing lines. This segmented architecture enables controlled bending of the aperture along the transverse direction, allowing the realization of prescribed cylindrical conformal profiles while preserving straight geometry and uniform spacing along the axial direction. The hinge angles are adjusted to approximate the target curvature, ensuring that the local orientation of each segment conforms to the desired cylindrical geometry. As illustrated by the renderings in Figure 5a, reconfiguring the hinge settings allows the same metasurface aperture to be transformed among planar, convex, concave, and convex‐concave configurations, thereby enabling a consistent experimental comparison of different curvature scenarios using a single hardware platform under otherwise identical EM and control conditions. Figure 5b shows a photograph of the actual prototype in the anechoic‐chamber experimental setup (see the Experimental Section for details). The segmented realization is consistent with the local‐planar modeling approach adopted in Section 2: each meta‐atom is described using the reflection response of a planar unit cell at the corresponding local incidence angle, and the local orientation of each segment is included in the synthesis. Thus, the piecewise‐linear approximation of the cylindrical profile is not neglected in the model. In the prototype, each rigid segment hosts one meta‐atom along the azimuthal direction, with a width of 14 mm, corresponding to about λ_*c*_/2 at *f_c_* =  10.3 GHz. Relative to an ideal continuously curved profile, segmentation therefore mainly discretizes the local surface normal on a subwavelength‐to‐half‐wavelength scale. For the moderate hinge angles considered here, this may slightly affect sidelobe and ringing levels but is not expected to produce appreciable beam‐pointing errors. Its effect is therefore treated as part of the implemented geometry rather than as an unmodeled fabrication error.

Figure S2 compares the measured and simulated reflection responses (magnitude and phase) of the meta‐atom, demonstrating good agreement.

Given the simplified, column‐controlled nature of the prototype, in which all meta‐atoms within a given column share a common bias signal, the experimental demonstration is constrained to quasi‐1D pattern syntheses and enforces invariance along the axial direction. Accordingly, we focus on the azimuthal synthesis problem in Equation (13) for fixed *θ*  =  90°. This limitation is associated with the present hardware implementation rather than with the synthesis framework itself: the formulation in Equations (13) and (14), together with the numerical examples in Figure 3 and Figure 4, assumes independent coding along both the conformal and axial directions and therefore provides numerical support for fully 2D synthesis. A corresponding experimental implementation would require independently addressable meta‐atoms, or an independently controlled row‐column architecture, following design principles already demonstrated in planar programmable/STC metasurfaces. Its realization in a conformal geometry is left for future work.

**FIGURE 6:**
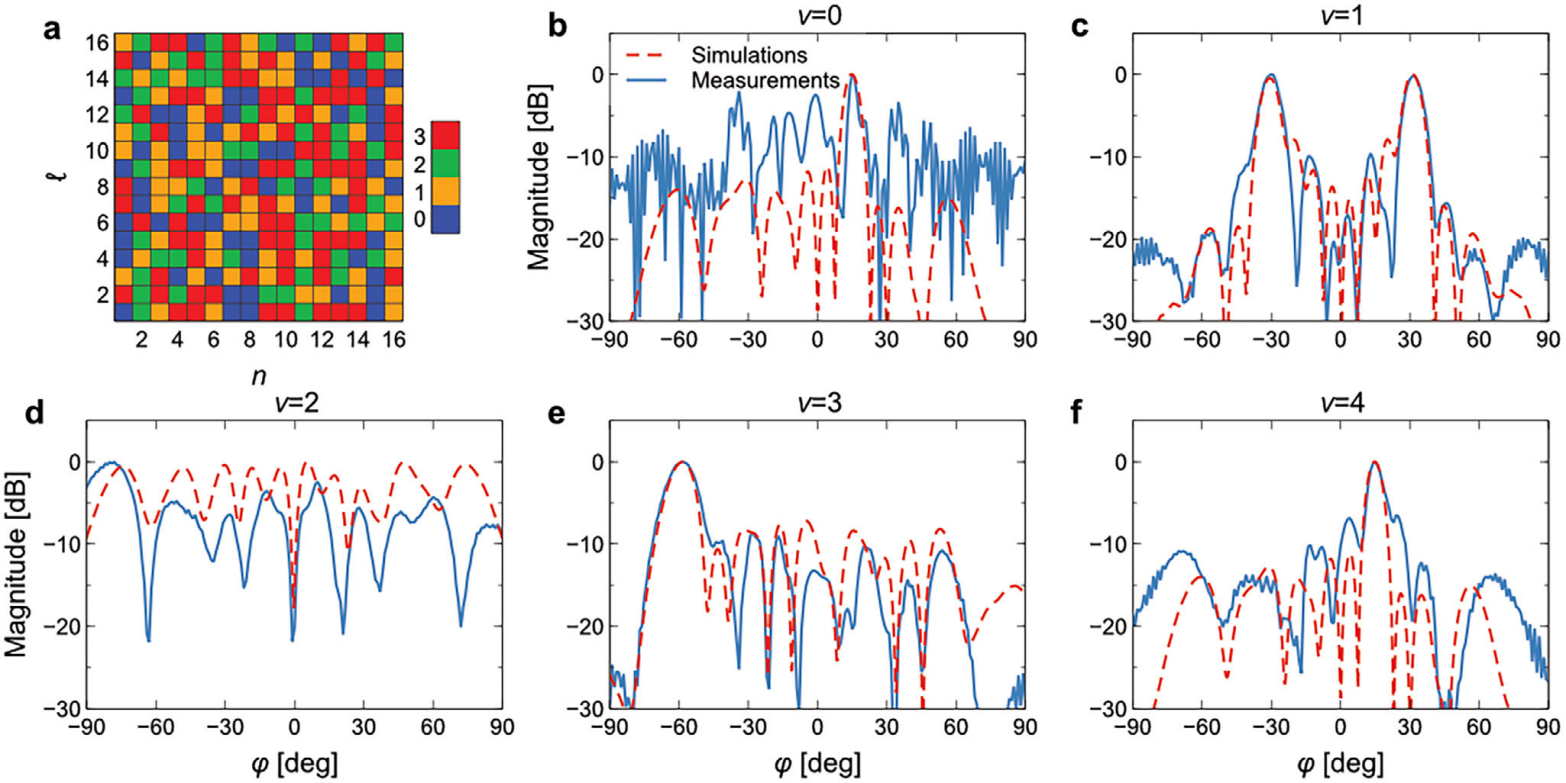
Experimental validation of multifrequency synthesis for a convex cylindrical configuration. Results correspond to Case *A* in Figure 2a, with *R*  =  300 mm and *D*  =  224 mm, assuming *f_c_* =  10.3GHz and *P*  =  *Q*  =  *S*  =  4, i.e., *L*  =  *PQ*  =  16. (a) STC matrix represented in a false‐color scale. (b–e) Comparison between simulated (dashed curves) and measured (solid curves) azimuthal scattering patterns at the harmonic orders ν  =  0,  1,  2, and 3, respectively, for fixed *θ*  =  90°. The synthesized responses correspond to an anomalous reflection at *φ*  =  15°, a beam‐splitting pattern at *φ*  =   ± 30°, a diffuse‐scattering pattern, and an anomalous reflection at *φ*  =   − 60°, respectively. Panel (f) additionally shows the scattering pattern at the harmonic order ν  =  4, which theoretically replicates, apart from a multiplicative constant, the response at ν  =  0. All patterns are normalized with respect to their maximum value.

Figure 6 presents the results for a first experimental example involving a convex conformal profile (Case *A* in Figure 2a, with *R*  =  300 mm and *D* = 224 mm). Figure 6a shows the STC matrix obtained by applying the frequency‐decoupling procedure outlined in Section 2 and synthesizing the individual harmonic patterns via evolutionary optimization (see the Experimental Section for details). In view of the assumed axial invariance, the STC matrix is displayed in a flattened, 2D form, indexed by *n* (space) and ℓ (time). As in the previous numerical examples, we assume *P*  =  *Q*  =  *S*  =  4, corresponding to a sequence length *L*  =  *PQ*  =  16, which enables independent control of the harmonic orders ν  =  0,  1,  2,  3. Figure 6b–e compares the simulated and measured azimuthal scattering patterns at these harmonic orders. The synthesized responses correspond to an anomalous reflection at *φ*  =  15°, a beam‐splitting pattern at *φ*  =   ± 30°, a diffuse‐scattering pattern, and an anomalous reflection at *φ*  =   − 60°, respectively. Overall, good agreement between simulations and measurements is observed. A moderately larger discrepancy, manifested by increased sidelobe levels and ringing, appears at the fundamental harmonic order ν  =  0. This behavior is attributed to the fact that the ν  =  0 response is measured at the same frequency as the incident carrier. Consequently, the received signal can include not only the metasurface‐reflected field, but also coherent contributions from residual feed leakage, feed‐antenna back radiation, blockage‐induced perturbations, and static chamber/background scattering, all occurring at *f_c_*. These carrier‐frequency contributions can interfere with the desired reflected field and are not fully captured by the simplified scattering model. By contrast, the up‐converted harmonic components are generated by the temporal modulation of the metasurface and are spectrally separated from the unmodulated carrier leakage and most static background contributions, which explains the generally better agreement observed for ν ≠ 0. For reference, Figure 6f reports the scattering pattern at ν  =  4, which theoretically replicates, apart from a multiplicative constant, the response at ν  =  0. The improved agreement observed at this harmonic supports the interpretation that the larger discrepancy at ν  =  0 is mainly related to carrier‐frequency feed/blockage and background effects rather than to a systematic error in the synthesized spatial pattern.

**FIGURE 7:**
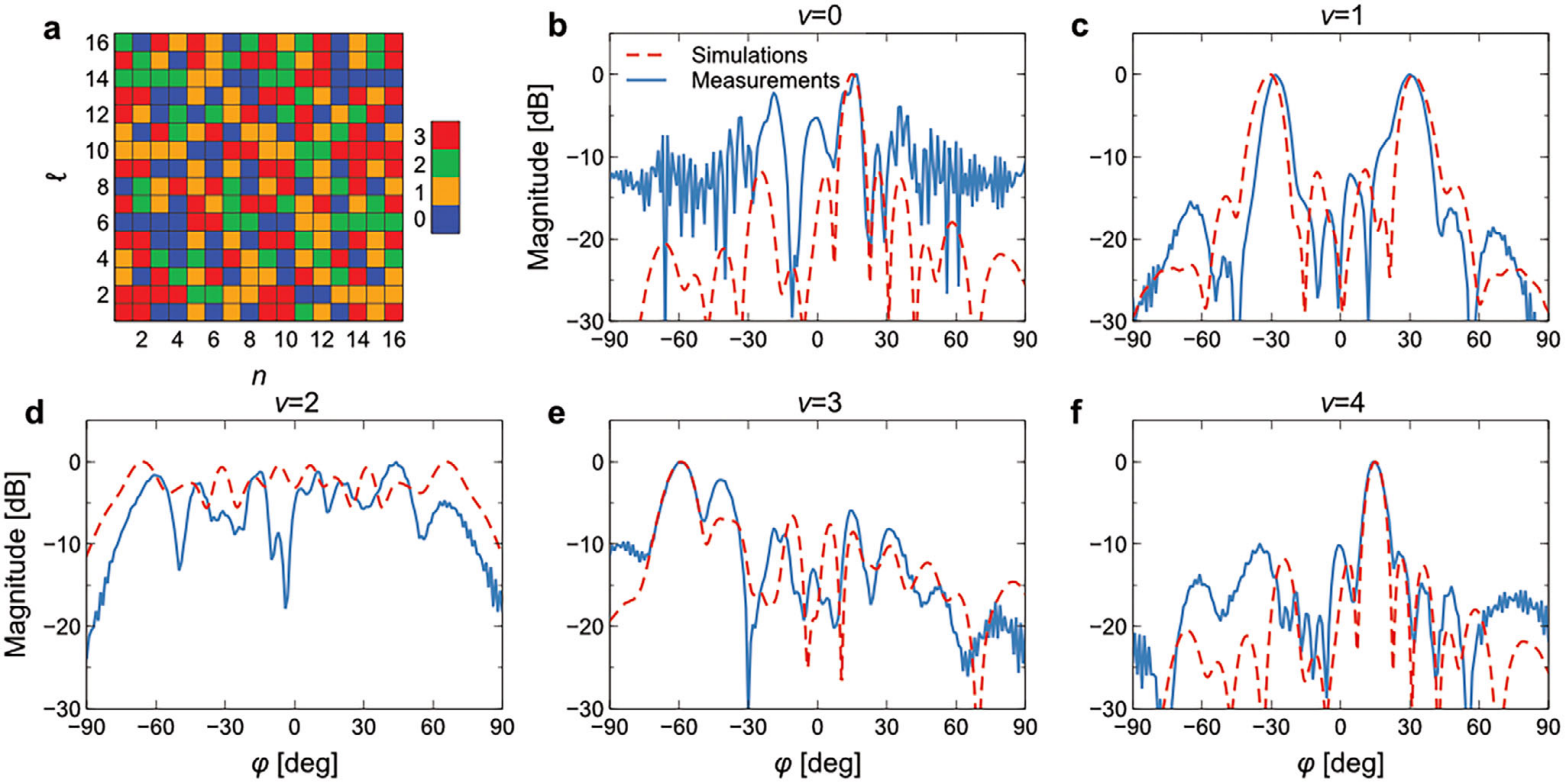
Experimental validation of multifrequency synthesis for a concave cylindrical configuration. Results correspond to Case *B* in Figure 2a, with *R*  =  300 mm and *D*  =  224 mm, assuming *f_c_* =  10.3 GHz and *P*  =  *Q*  =  *S*  =  4, i.e., *L*  =  *PQ*  =  16. (a) STC matrix represented in a false‐color scale. (b–e) Comparison between simulated (dashed curves) and measured (solid curves) azimuthal scattering patterns at the harmonic orders ν  =  0,  1,  2, and 3, respectively, for fixed *θ*  =  90°. The synthesized responses correspond to an anomalous reflection at *φ*  =  15°, a beam‐splitting pattern at *φ*  =   ± 30°, a diffuse‐scattering pattern, and an anomalous reflection at *φ*  =   − 60°, respectively. Panel (f) additionally shows the scattering pattern at the harmonic order ν  =  4, which theoretically replicates, apart from a multiplicative constant, the response at ν  =  0. All patterns are normalized with respect to their maximum value.

Figure 7 presents the results for a concave conformal profile (Case *B* in Figure 2a, with *R*  =  300mm and *D*  =  224mm), considering the same set of synthesized harmonic scattering patterns as in the previous example. Qualitatively, the observed behavior is similar to that discussed above. This example serves a twofold purpose. First, it demonstrates that the proposed approach enables the synthesis of prescribed harmonic scattering patterns independently of the specific conformal geometry. Second, the markedly different structures of the STC matrices in Figures 6a and 7a clearly indicate that the synthesis outcome is strongly dependent on the underlying surface shape. To further emphasize this critical aspect, Figure S3 illustrates the consequences of neglecting curvature effects by assuming a planar structure during the STC design. As shown, applying such a planar‐based design to a curved metasurface leads to completely inaccurate scattering responses.

**FIGURE 8:**
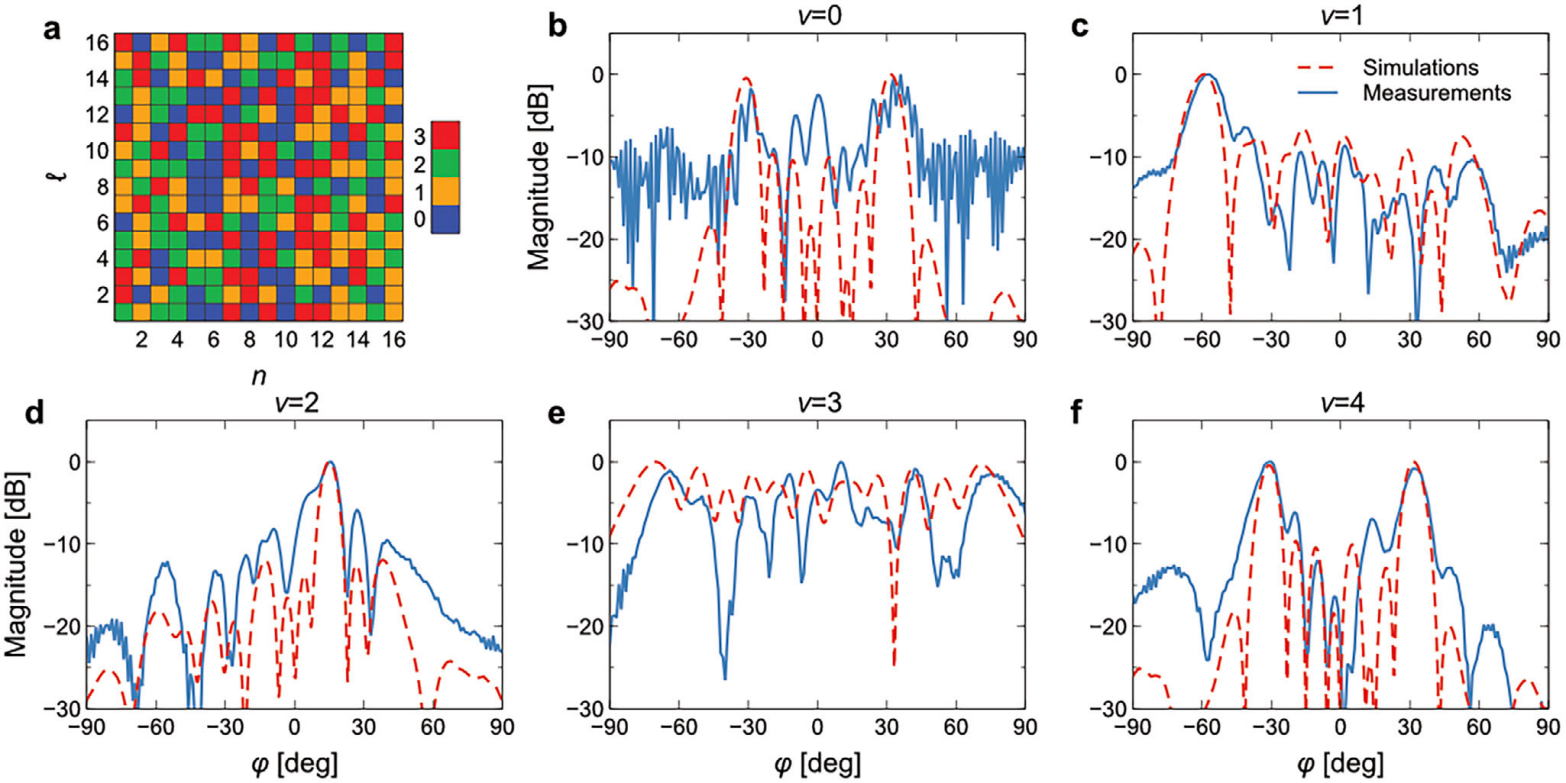
Experimental validation of multifrequency synthesis for a convex‐concave cylindrical configuration. Results correspond to Case *C* in Figure 2a, with *R*  =  150 mm and *D*  =  224 mm, assuming *f_c_* =  10.3 GHz and *P*  =  *Q*  =  *S*  =  4, i.e., *L*  =  *PQ*  =  16. (a) STC matrix represented in a false‐color scale. (b–e) Comparison between simulated (dashed curves) and measured (solid curves) azimuthal scattering patterns at the harmonic orders ν  =  0,  1,  2, and 3, respectively, for fixed *θ*  =  90°. The synthesized responses correspond to a beam‐splitting pattern at *φ*  =   ± 30°, an anomalous reflection at *φ*  =   − 60°, an anomalous reflection at *φ*  =  15°, and a diffuse‐scattering pattern, respectively. Panel (f) additionally shows the scattering pattern at the harmonic order ν  =  4, which theoretically replicates, apart from a multiplicative constant, the response at ν  =  0. All patterns are normalized with respect to their maximum value.

Finally, Figure 8 presents the results for a convex‐concave conformal profile (Case *C* in Figure 2a, with *R*  =  150mm and *D*  =  224mm). In this case, we consider the same set of scattering patterns synthesized in the previous examples, but redistributed among the harmonic orders to further illustrate the flexibility and multiplexing capabilities of the proposed approach. Qualitatively, the observed behavior is consistent with those discussed above. The validity of the local‐planar physical‐optics approximation is most demanding in regions where the surface orientation varies rapidly. In particular, for the convex‐concave profile of Case *C*, the transition region between the two opposite curvatures can, in principle, introduce additional diffraction, local scattering, and nonlocal coupling effects that are not fully captured by the semi‐analytical model. For the moderate curvature considered here, however, these effects appear to remain limited, as indicated by the overall good agreement between simulations and measurements. Residual discrepancies are expected to mainly affect sidelobe and ringing levels rather than the prescribed main‐beam directions.

Overall, these examples validate the proposed methodology in a realistic experimental setting and highlight its relevance for practical applications.

## 4 Conclusions

In this work, we have demonstrated STC conformal metasurfaces as an effective platform for dynamic EM wave control on curved structures. By extending STC concepts to conformal geometries, we have shown that a broad class of harmonic scattering responses can be independently synthesized at different harmonic orders using a single, nonplanar aperture, as exemplified by beam steering, beam splitting, and diffuse scattering. We have adopted a cylindrical framework that captures curvature effects while enabling accessible modeling and hybrid synthesis strategies combining semi‐analytical design and evolutionary optimization. Experimental validation using an X‐band programmable prototype mounted on a mechanically reconfigurable support shows good agreement with simulations across multiple harmonic orders and conformal profiles, confirming the feasibility of STC on realistic curved platforms.

From a communications and RIS perspective, these results point toward multifunctional conformal surfaces capable of frequency‐multiplexed beam control, dynamic spectrum utilization, and geometry‐aware wave shaping. More broadly, the proposed framework may provide a useful basis for other emerging conformal EM functionalities. For example, geometry‐aware control on curved apertures is relevant to conformal cloaking and dynamic illusion concepts [67], while the harmonic‐domain control enabled by STC may be exploited for EM deception, radar camouflage, and radar‐signature manipulation [68]. Likewise, the ability to assign different scattering responses to different harmonic frequencies is naturally connected to STC‐enabled space‐frequency‐division multiplexing for wireless communications [69]. Extensions to these scenarios would require application‐specific target functions and spectral‐efficiency constraints.

Future work will address fully 2D experimental control using independently addressable conformal apertures, more general conformal geometries, and strongly curved or electrically small platforms requiring higher‐fidelity modeling beyond the present physical‐optics approximation. In particular, implementing fully independent 2D control on conformal hardware will require addressing several engineering challenges, including high‐density via fabrication, reliable routing of direct‐current bias lines on flexible substrates such as polyimide, and mechanical robustness under bending. Recent advances in flexible and stretchable EM materials and devices may provide promising routes to overcome these constraints and enable alternative conformability technologies [70]. Further directions include advanced temporal coding schemes aimed at improved spectral selectivity, replica suppression, and scalability, as well as integration into practical platforms such as unmanned aerial vehicles.

## 5 Experimental Section

### 5.1 Modeling and Design

The meta‐atom shown in Figure 2b is modeled and designed using the finite‐element‐based commercial software Ansys HFSS [71]. The unit cell has an overall size of 14 × 14 mm^2^ and consists of a hexagonal copper patch connected to two metallic strips through two PIN diodes. The strips are symmetrically arranged with respect to the unit‐cell center and serve as DC bias feed lines for diode control. The structure is implemented on a 1.5‐mm‐thick F4B dielectric substrate backed by a copper ground plane, with a nominal relative permittivity ε_*r*_ =  2.6 and loss tangent tan δ =  0.001. To facilitate conformal integration, 0.5‐mm‐wide slots are etched on both lateral sides of the unit cell. The 0.2‐mm‐thick polyimide (PI) film shown in Figure 2b is included solely for mechanical conformability and is not modeled in the EM simulations. The PIN diodes (MACOM MADP‐000907‐14020x) are represented by lumped‐element series models, consisting of an RL network (3.35Ω, 200pH) in the ON state and an RLC network (2Ω, 200pH, 52fF) in the OFF state. To emulate an infinite periodic array, the unit cell is truncated using phase‐shift (Floquet) boundary conditions in the *y* − *z* plane. Excitation is provided by a wave port located above the unit cell, generating an obliquely incident plane wave with *z*‐polarized electric field. Scattering parameters are extracted for all four combinations of the diode states.

An adaptive tetrahedral meshing strategy is employed, with convergence enforced through a maximum delta‐energy criterion of 0.02 and a minimum of 25 adaptive passes. The mesh is refined down to λ_c_/3, resulting in approximately 150000 elements upon convergence.

After optimization, the meta‐atom exhibits a 2‐bit phase response with nearly 90° phase steps and low reflection loss at the operating frequency 10.3 GHz for all diode‐state combinations, as shown in Figure 2c. In these simulations, a slightly reduced permittivity value ε_*r*_ =  2.4 is assumed to achieve improved agreement with measurements (see Figure S1), a deviation well within the material uncertainty of F4B. The angular response characteristics reported in Figure 2d are obtained by varying the incidence angle at the design frequency of 10.3 GHz.

For the synthesis of the azimuthal scattering patterns, an evolutionary optimization strategy is adopted, implemented through an in‐house genetic‐algorithm code developed in MATLAB [72]. For the anomalous‐reflection case, the optimization minimizes the maximum scattered‐field magnitude outside the prescribed angular window. For the beam‐splitting case, it minimizes the ratio between the maximum scattered‐field magnitude outside the prescribed angular windows and the average of the maximum values within the prescribed windows. For the diffuse‐scattering case, the objective function aims to minimize the crest factor of the scattering pattern.

The genetic algorithm (GA) operates with a population size of 1000 individuals and a maximum of 200 generations, using a crossover probability of 0.9 and a mutation probability of 0.05. Owing to the frequency‐decoupling procedure, the GA is not applied to the full STC matrix. For the 1D experimental syntheses, each run optimizes only *N*  =  16 spatial coding coefficients for a single harmonic order, each taking one of *S*  =  4 states, so that the relevant search space is 4^16^. The *N* × *L* STC matrix is subsequently assembled by analytical interleaving of the optimized subsequences. Typical convergence required 100–150 generations, corresponding to approximately 1.0 × 10^5^ − 1.5 × 10^5^ objective‐function evaluations per harmonic, with a wall‐clock time of about 45–60 minutes on a workstation equipped with an Intel Xeon E5‐1607 v4 processor operating at 3.10 GHz, 4 physical cores/4 logical processors, and 64 GB RAM.

### 5.2 Prototype Realization

The fabricated prototype, based on the meta‐atom design described above, comprises 16 columns, each formed by 8 interconnected unit cells. For simplicity of realization, all meta‐atoms within a given column share a common DC bias voltage, with a 1‐mm‐wide slot separating adjacent columns. Each column is fabricated individually using standard printed circuit board processes on a 1.5‐mm‐thick F4B dielectric substrate, then connected through precision hinges and assembled onto a 0.2‐mm‐thick PI film laminated beneath the substrate. This flexible supporting layer enhances conformal integration, enabling controlled bending and deployment on curved or flexible platforms. Bias voltages are supplied by an FPGA‐based control board (ALTERA Cyclone IV EP4CE6) preloaded with the desired STC matrices. When fully flattened, the prototype has overall dimensions of 224 × 122 mm^2^, corresponding to an effective reflective aperture of 224 × 112 mm^2^. A modulation frequency of *f*_0_ =  1 MHz is employed, corresponding to a PIN‐diode switching rate of  *f_s_* =  16 MHz. This modulation frequency sets the spacing between adjacent STC harmonics and was selected for practical compatibility with the FPGA controller and PIN‐diode switching dynamics. It should be distinguished from the carrier‐frequency operating bandwidth discussed in Section 3.1: larger harmonic spacings would require faster switching electronics, whereas broader carrier‐frequency operation would require a broadband meta‐atom design.

### 5.3 Measurements

The reflection response of the individual meta‐atom (Figure S1) is characterized using a Keysight N5230C vector network analyzer (VNA). Far‐field measurements of the harmonic scattering patterns are performed in a standard microwave anechoic chamber, as shown in Figure 5b. A linearly polarized X‐band horn antenna is used as the plane‐wave excitation source, while a broadband horn antenna connected to a VNA serves as the receiver. The feed antenna and the metasurface prototype are coaxially mounted on rotating arms separated by 0.85 m, allowing full 360° rotation in the horizontal plane with an angular resolution of 0.1°. As illustrated in Figure 5, the metasurface prototype is mechanically deformed into three different conformal profiles (Cases *A*, *B*, and *C*) using a 3D‐printed plastic support. The corresponding parametric descriptions of the profiles are provided in the Supporting Information. Harmonic scattering patterns are measured for each conformal configuration.

## Conflicts of Interest

The authors declare no conflicts of interest.

## Supporting information




**Supporting File 1**: adma73837‐sup‐0001‐SuppMat.pdf.

**Supporting File 2**: adma73837‐sup‐0002‐3STCM‐Figure3.txt.

**Supporting File 3**: adma73837‐sup‐0003‐3STCM‐Figure4.txt.

## Data Availability

The data that support the findings of this study are available from the corresponding author upon reasonable request.
