# Supplementary material for: Space‐Time Coding Conformal Metasurfaces for Multifrequency Beam Steering and Shaping
Source: Adv Mater. 2026 Jul 7;38(45):e73837. doi: 10.1002/adma.73837 (PMC13471822; doi:10.1002/adma.73837)
Supplement: Supplementary file 1 [file ADMA-38-e73837-s001.pdf]

Supporting Information

Space-Time Coding Conformal Metasurfaces for Multifrequency Beam Steering and Shaping

Filippo Pepe[†]*, Lei Zhang*[†,*]*, Yi Ning Zheng, Xiao Qing Chen, Ivan Iudice, Giuseppe Castaldi, Marco Di Renzo, Tie Jun Cui*[*]*, and Vincenzo Galdi*[*]

This document contains additional details and results on the modeling and design. Newly introduced equations, references and figures are labeled with the prefix "S"; all others pertain to the main text.

Details on Semi-Analytical Modeling

Referring to the schematic in Figure 1, we consider a cylindrical conformal metasurface described by a 3D surface Ω, parameterized as

$$\boldsymbol{r}_{\Omega}(u,z) = X(u)\hat{\boldsymbol{x}} + Y(u)\hat{\boldsymbol{y}} + z\hat{\boldsymbol{z}}, \qquad 0 \le u \le 1, \qquad 0 \le z \le D_z, \tag{S1}$$

where $\{X(u), Y(u)\}$ defines the conformal profile in the $x-y$ plane. The metasurface is illuminated by a time-harmonic plane wave with implicit $\exp(j2\pi f_c t)$ dependence, unit amplitude, and z-polarized electric field, impinging from the negative x-direction,

$$\boldsymbol{E}^i(x) = \exp(jk_c x)\,\hat{\boldsymbol{z}}\,. \tag{S2}$$

At this stage, temporal modulation is neglected. To model the scattered field in the $x > 0$ half-space in a simple yet effective manner, we adopt the physical-optics (PO) approximation [S1].

Specifically, we employ the electric-field formulation [S2], which approximates scattering from a smoothly curved reflecting surface by locally treating it as planar and radiating equivalent magnetic surface currents,

$$K_{PO}(\boldsymbol{r}_\Omega) = 2\Gamma(\boldsymbol{r}_\Omega)\boldsymbol{E}^i(X) \times \hat{\boldsymbol{n}}, \tag{S3}$$

where $\Gamma(\boldsymbol{r}_\Omega)$ denotes the local reflection coefficient for the electric field, and

$$\hat{\boldsymbol{n}}(u) = \frac{\dot{X}(u)\,\hat{\boldsymbol{y}} - \dot{Y}(u)\,\hat{\boldsymbol{x}}}{\sqrt{\dot{X}^2(u) + \dot{Y}^2(u)}} \tag{S4}$$

is the local outward unit normal vector to the conformal profile, with the overdot denoting differentiation with respect to the argument. Accordingly, the scattered electric field radiated into the $x > 0$ half-space can be expressed through the canonical radiation integral [S3]

$$\boldsymbol{E}^s(\boldsymbol{r}) = 2\nabla \times \iint_\Omega \hat{\boldsymbol{n}} \times \boldsymbol{E}^i(X)\,\Gamma(\boldsymbol{r}_\Omega)G(\rho)d\boldsymbol{r}_\Omega, \tag{S5}$$

where

$$G(\rho) = \frac{\exp(-jk_c\rho)}{4\pi\rho} \tag{S6}$$

is the 3D scalar Green's function, with $\rho = |\boldsymbol{r} - \boldsymbol{r}_\Omega|$, and the differential surface element can be expressed as $d\boldsymbol{r}_\Omega = \sqrt{\dot{X}^2(u) + \dot{Y}^2(u)}dudz$.

In the far-field region, Equation (S5) can be asymptotically approximated as

$$\begin{aligned}\boldsymbol{E}^s_\infty(\boldsymbol{r}) \sim \frac{jk_c\exp(-jk_c r)}{2\pi r}\int_0^1 du\int_0^{D_z} dz\,\exp[jk_c X(u)]\,\exp[j\boldsymbol{k}(\theta,\varphi)\\ \cdot\,\boldsymbol{r}_\Omega(u,z)]\Gamma(\boldsymbol{r}_\Omega)\{-\dot{Y}(u)\cos\theta\hat{\boldsymbol{x}} + \dot{X}(u)\cos\theta\hat{\boldsymbol{y}}\\ + \sin\theta[\dot{Y}(u)\cos\varphi - \dot{X}(u)\sin\varphi]\hat{\boldsymbol{z}}\}.\end{aligned} \tag{S7}$$

By discretizing the surface into $N \times M$ subwavelength pixels (meta-atoms), the integral in Equation (S7) can be further approximated as

$$\boldsymbol{E}^{s}_{\infty}(\boldsymbol{r})$$

$$\approx \frac{jk_c \exp(-jk_c r)}{2\pi r} \sum_{n=1}^{N} \sum_{m=1}^{M} \Gamma_{nm} \exp(jk_c x_n) \exp[j\boldsymbol{k}(\theta,\varphi)\cdot \boldsymbol{r}_{nm}][-\dot{y}_n \cos\theta \hat{\boldsymbol{x}} \quad \text{(S8)}$$

$$+ \dot{x}_n \cos\theta \hat{\boldsymbol{y}} + \sin\theta(\dot{y}_n \cos\varphi - \dot{x}_n \sin\varphi)\hat{\boldsymbol{z}}]\Delta\Omega.$$

Here, Γ_{nm} denotes the locally sampled reflection coefficient, assumed uniform within each meta-atom, $\Delta\Omega$ is proportional to the area of a meta-atom, assumed identical across the surface, and all other symbols are defined in the main text. If we now introduce a temporal modulation of the local reflection coefficients on a time scale *much slower* than the EM period, Equation (S8) can be assumed to hold *adiabatically* by introducing a time-varying reflection coefficient $\Gamma_{nm}(t)$. Equation (5) in the main text then follows by rewriting the vector quantities in spherical components, explicitly introducing the time-harmonic dependence, and neglecting irrelevant constants as well as the dependence on the observation distance *r*.

Details on Multifrequency Synthesis Approach

The joint multifrequency synthesis strategy adopted in this work extends to conformal geometries the analytical framework originally developed for planar metasurfaces in [S4]. For completeness, the main elements of this strategy are outlined below.

Since the synthesis procedure is applied independently to each meta-atom, we simplify the notation by omitting the subscript (n,m) and consider a representative element. The goal is to control a set of Q harmonic frequencies, corresponding to harmonic orders $\nu = 0, 1, \ldots, Q-1$. The overall temporal coding sequence is organized into Q interleaved sub-sequences, each consisting of P elements, yielding a total sequence length $L = PQ$. To impose a structured relationship among the harmonics, we enforce a symmetry condition on the reflection-coefficient sequence. Specifically, the sequence elements satisfy

$$\Gamma^{(q+1+pQ)} = \Lambda^{(pq)} \Gamma^{(q+1)}, \qquad p = 0,\ldots,P-1, \qquad q = 0,\ldots,Q-1\,. \qquad \text{(S9)}$$

Here, $\Lambda^{(pq)}$ are phase-shift factors selected from the same quantized set as the reflection-coefficient states, so that the resulting products remain within the admissible set. For self-consistency, we impose $\Lambda^{(0q)} = 1$ for all $q = 0, \dots, Q-1$. It is worth noting that the exact factorization expressed in Equation (S9) is rigorously valid only in the idealized case of uniform phase spacing among the reflection-coefficient states, as occurs, for example, in a 2-bit implementation with 90° phase increments. In the conformal configuration considered here, however, the set of accessible reflection-coefficient states varies across the metasurface as a consequence of the locally varying incidence direction (see also the discussion in the main text). Despite this, provided that the relative phase differences between the available states remain sufficiently insensitive to the incidence angle, the symmetry condition in Equation (S9) can still be regarded as a good approximation. As demonstrated below, the meta-atom employed in this study exhibits an angular response that fulfills this requirement to a satisfactory degree.

By enforcing the symmetry constraint in Equation (S9), the expression for the ν-th harmonic coefficient in Equation (7) can be recast as

$$a^{(\nu)} = c^{(\nu)} \sum_{q=0}^{Q-1} \Gamma^{(q+1)} \alpha^{(q\nu)} \exp\left(\frac{-j\pi\nu q}{L}\right), \tag{S10}$$

where

$$c^{(\nu)} = \frac{1}{L} \operatorname{sinc}\left(\frac{\pi\nu}{L}\right) \exp\left(-\frac{j\pi\nu}{L}\right) \tag{S11}$$

is a frequency-dependent factor that determines the overall decay of the harmonic spectrum. The quantities

$$\alpha^{(q\nu)} = \sum_{p=0}^{P-1} \Lambda^{(pq)} \exp\left(-\frac{j2\pi\nu p}{P}\right) \tag{S12}$$

play the role of digital filter coefficients. By appropriately selecting the phase-shift factors $\Lambda^{(pq)}$, these coefficients can be engineered to shape the harmonic content in a controlled manner. In the ideal case of fully decoupled synthesis, they should satisfy

$$\alpha^{(qv)} \propto \tilde{\delta}_{qv}, \tag{S13}$$

where $\tilde{\delta}_{qv}$, denotes a reduced Kronecker delta defined as

$$\tilde{\delta}_{qv} = \begin{cases} 1, & q = v \\ 0, & q \neq v \end{cases}, \qquad q, v = 0, \ldots, Q-1. \tag{S14}$$

This condition ensures that each of the Q interleaved sub-sequences excites a single harmonic order, enabling independent and simultaneous control of the targeted spectral components.

As shown in [S4], a particularly convenient closed-form solution arises when the number of controlled harmonic frequencies Q does not exceed the number of available quantized reflection-phase states S. In this case, choosing $P = S$ and defining the phase-shift factors as

$$\Lambda^{(pq)} = \exp\left(\frac{j2\pi pq}{S}\right), \tag{S15}$$

corresponding to quantized linear phase progressions, yields

$$\alpha^{(qv)} = Q\tilde{\delta}_{qv}. \tag{S16}$$

thus realizing the desired filtering behavior. Substitution of this result into Equation (S10), together with the property in Equation (S14), directly leads to Equation (9) in the main text.

In the numerical examples presented in this work, a 2-bit configuration is considered, with $S = Q = P = 4$. Under these conditions, each filter coefficient $\alpha^{(qv)}$ selectively addresses one of the four targeted harmonic orders. Owing to the finite length of the sub-sequences, the resulting response does not correspond to an ideal Kronecker delta, but instead exhibits a periodic structure with period Q. Consequently, the synthesized scattering patterns associated with the first Q harmonic orders repeat at higher orders, with amplitudes progressively attenuated according to the spectral envelope imposed by the factor $\xi^{(v)}$ in Equation (10). By symmetry, analogous repetitions occur for negative harmonic orders.

As discussed in [S4] and in the main text, a more elaborate temporal coding can be employed to further tailor the spectral roll-off, effectively implementing a low-pass digital filtering action on the harmonic spectrum.

Finally, the maximum number of independently controllable harmonic orders is constrained by both the number of available phase states S and the switching rate f_s of the active elements. Assuming $P = S$ to retain analytical tractability, the overall modulation period is $T_0 = SQT_s$, where $T_s = 1/f_s$ is the switching interval. The corresponding modulation frequency is therefore $f_0 = f_s/(SQ)$. For a given minimum required harmonic spacing $f_{0,min}$, dictated by application-specific constraints and spectral resolution, the maximum number of addressable harmonic orders is given by $Q_{\max} = f_s/(Sf_{0,\min})$.

Impact of the Factorization and Projection Approximation

The synthesis strategy in the main text relies on the approximate factorization of the reflection coefficients in Equation (11), followed by the projection onto the locally admissible reflection states through Equation (15). To illustrate the impact of these approximations, **Figure S1** compares the scattering patterns in Figure 3 obtained before and after the projection step. Specifically, the ideal factorized pattern is computed using the product of the independently synthesized conformal-direction and axial-direction coefficients, whereas the implementable pattern is obtained after projecting this product onto the available 2-bit reflection states of each meta-atom.

As observed, the factorization/projection step preserves the prescribed main-beam direction and the overall morphology of the synthesized response. The main differences are confined mainly to sidelobe regions and fine pattern details, which is expected because the projection locally quantizes the ideal factorized coefficients according to the admissible set $\boldsymbol{\Xi}_{\boldsymbol{n}}$. These results support the use of the factorized synthesis for the canonical beam-steering and beam-shaping functionalities considered in this work, while also highlighting that the approximation is not intended as an exact decomposition for arbitrary 2D scattering patterns.

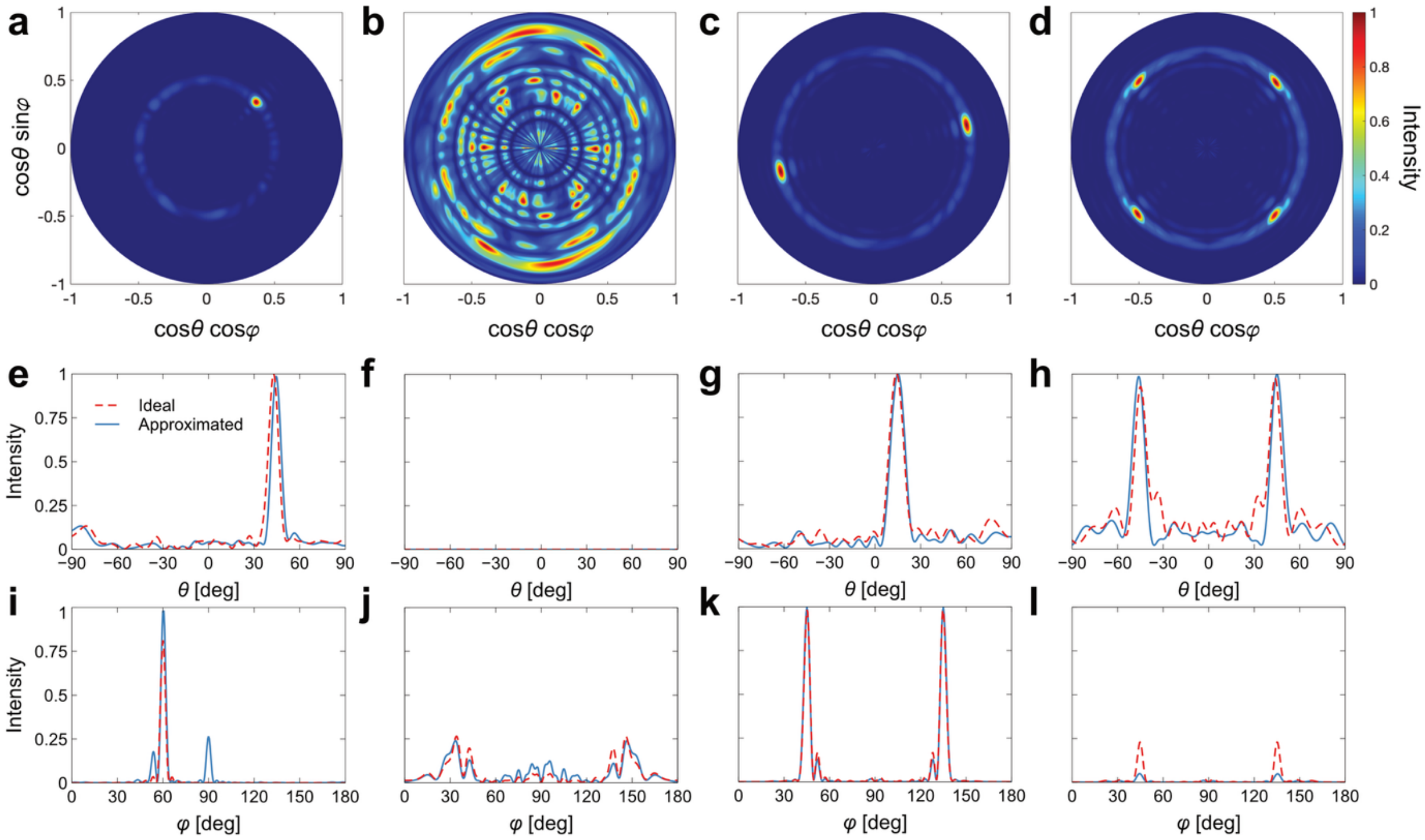

Figure S1 | Impact of the factorization/projection approximation. a–d) Ideal factorized patterns for the configuration in Figure 3 of the main text, obtained from the product of the independently synthesized conformal-direction and axial-direction coefficients before projection. e–h) Comparison between ideal and approximated (projected) patterns along representative cuts at $\boldsymbol{\varphi = 45°, 0°, 15°, 30°}$, respectively. i–l) Comparison between ideal (factorized) and approximated (projected) patterns along representative cuts at $\boldsymbol{\theta = 60°, 90°, 45°, 45°}$, respectively.

Parametric Equations of Conformal Profiles Considered in the Study

For completeness and reproducibility, we report here the parametric equations of the three conformal profiles considered throughout this study. All profiles are defined in the $x - y$ plane as functions of a normalized parameter $u \in [0,1]$.

Case A (convex cylindrical profile):

$$\begin{cases} X(u) = R\left\{\cos\left(\frac{D}{2R}\right) - \cos\left[\frac{D}{2R}(1-2u)\right]\right\}, \\ Y(u) = R\left\{\sin\left[\frac{D}{2R}(1-2u)\right] + \sin\left(\frac{D}{2R}\right)\right\}. \end{cases} \quad \text{(S17)}$$

Case B (concave cylindrical profile):

$$\begin{cases} X(u) = R\left\{\cos\left[\frac{D}{2R}(1-2u)\right] - \cos\left(\frac{D}{2R}\right)\right\}, \\ Y(u) = R\left\{\sin\left[\frac{D}{2R}(1-2u)\right] + \sin\left(\frac{D}{2R}\right)\right\}. \end{cases} \quad \text{(S18)}$$

Case C (convex-concave profile):

$$\begin{cases} X(u) = \operatorname{sign}\left(u - \frac{1}{2}\right) R\left\{\cos\left(\frac{D}{4R}\right) - \cos\left[\frac{D}{2R}\left(u - \frac{1}{2}\right)\right]\right\}, \\ Y(u) = R\left\{\sin\left[\frac{D}{2R}\left(u - \frac{1}{2}\right)\right] + 2\sin\left(\frac{D}{4R}\right)\right\}. \end{cases} \quad \text{(S19)}$$

For all examples reported in Figures 3, 4, 6, 7, and 8, the parameters R and D are explicitly specified in the corresponding figure captions. The curvilinear abscissa (arc-length) associated with each profile can be computed as:

$$s(u) = \int_0^u \sqrt{\dot{X}^2(\tau) + \dot{Y}^2(\tau)}\, d\tau. \quad \text{(S20)}$$

Comparison Between Simulated and Measured Meta-Atom Responses

Figure S2 compares the simulated and measured reflection responses (magnitude and phase) of the 2-bit meta-atom shown in Figure 2b, for the four possible combinations of the PIN-diode states. As observed, the reflection magnitude is moderately overestimated in the simulations, yet remains within acceptable limits. More importantly, the phase response, which is critical for the intended STC operation, shows improved agreement with the measurements when a slightly reduced relative permittivity value ($\varepsilon_r = 2.4$, Figure S2a) is assumed in the simulations, compared to the nominal value ($\varepsilon_r = 2.6$, Figure S2b). This discrepancy, within approximately 10%, falls well within the typical parameter uncertainty associated with the F4B substrate.

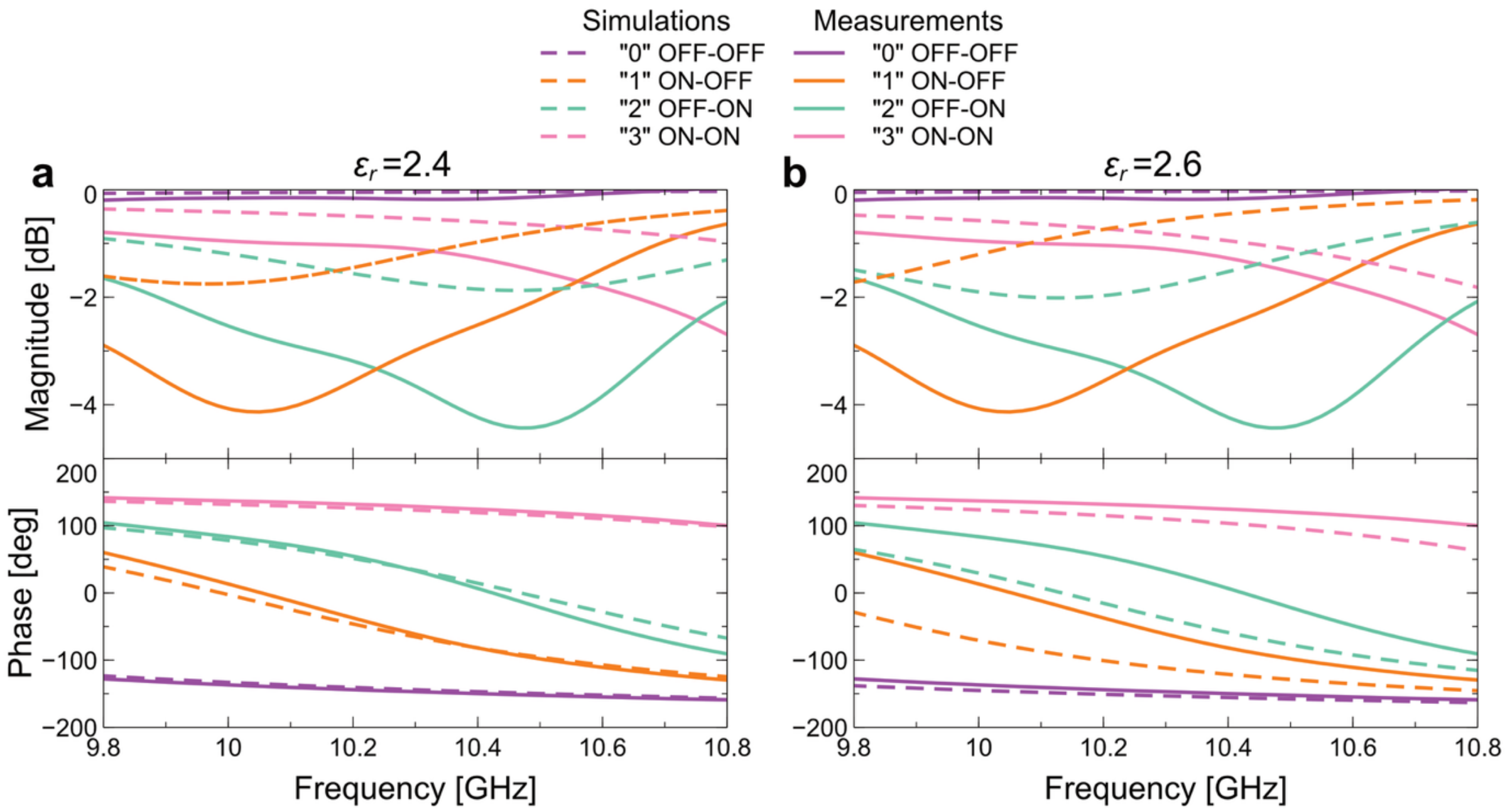

Figure S2 | a,b) Comparison between simulated (dashed curves) and measured (solid curves) frequency response of meta-atom (reflection-coefficient magnitude and phase) under normal incidence, assuming $\varepsilon_r = 2.4$ and $\varepsilon_r = 2.6$, respectively, for the F4B dielectric substrate.

Details on Golay-Rudin-Shapiro Polynomials

In the diffuse-scattering syntheses reported in Figures 3 and 4 for the main text, the axial synthesis in Equation (14) is carried out analytically by resorting to Golay-Rudin-Shapiro (GRS) polynomials [S5,S6]. These are well-known flat polynomials on the unit circle, characterized by low crest factors, and have been shown to provide effective sub-optimal analytical solutions in low-observability scenarios [S7]. The GRS polynomials can be defined recursively as:

$$P_{k+1}(x) = P_k(x) + x^{2^k}\, Q_k(x),$$
$$Q_{k+1}(x) = Q_k(x) - x^{2^k}\, P_k(x), \tag{S21}$$

with initialization $P_0(x) = Q_0(x) = 1$. It can be verified that the polynomials P_k and Q_k have 2^k coefficients, denoted by $p_{k,m}$ and $q_{k,m}$, respectively, each taking values ± 1. These coefficients can be generated explicitly as

$$p_{k,0} = 1, \qquad p_{k,2i} = p_{k,i}, \qquad p_{k,2i+1} = (-1)^i\, p_{k,i}, \tag{S22}$$

$$q_{k,i} = \begin{cases} p_{k,i}, & i = 0, \dots, 2^{k-1} - 1, \\ -p_{k,i}, & i = 2^{k-1}, \dots, 2^k - 1. \end{cases} \tag{S23}$$

In the synthesis examples considered in this work, we assume $M = 32$ elements along the axial direction and select the reflection coefficients in Equation (14) according to the coefficients of the Q_5 GRS polynomial, i.e., $\hat{\Gamma}_m^{(\nu+1)} = q_{5,m-1},\ m = 1, \dots, M$.

Algorithmic Summary of STC Matrix Generation

For reproducibility, we summarize here the main steps used to generate the STC matrices considered in this work. The procedure combines the frequency-decoupling strategy described in the main text with separate spatial syntheses along the conformal and axial directions, followed by projection onto the locally admissible reflection states and temporal interleaving.

Input: conformal profile $\{X(u), Y(u)\}$, number of meta-atoms N and M, coding alphabet {0,1,2,3}, number of controlled harmonics Q, subsequence length $P = S$, total sequence length $L = PQ$, local admissible reflection sets Ξ_n, and target functionality assigned to each harmonic order $\nu = 0, \ldots, Q-1$.

1. Compute the position $\boldsymbol{r}_{nm}$ and local orientation of each meta-atom from the prescribed conformal profile.
2. Determine the local incidence angle of each conformal-position index n, and assign the corresponding admissible reflection-coefficient set Ξ_n from the full-wave unit-cell simulations.
3. For each controlled harmonic order $\nu = 0, \ldots, Q-1$:
    a. Synthesize the conformal-direction coefficients $\tilde{\Gamma}_n^{(\nu+1)}$. For anomalous-reflection and beam-splitting responses, this is done using the genetic algorithm described in the Experimental Section. For diffuse-scattering responses, the genetic algorithm minimizes the crest factor of the corresponding scattering pattern.
    b. Synthesize the axial coefficients $\hat{\Gamma}_m^{(\nu+1)}$. For the canonical examples considered here, these coefficients are obtained analytically using linear phase profiles, binary beam-splitting profiles, or GRS sequences, depending on the target functionality.
    c. Form the ideal factorized coefficient $\Gamma_{nm}^{(\nu+1),fac} = \tilde{\Gamma}_n^{(\nu+1)} \hat{\Gamma}_m^{(\nu+1)}$.
    d. Project the ideal coefficient onto the locally admissible reflection states $\Gamma_{nm}^{(\nu+1),impl} = \mathcal{P}_{\Xi_n}[\Gamma_{nm}^{(\nu+1),fac}]$, where $\mathcal{P}_{\Xi_n}$ is the projection operator defined in Equation (16) of the main text.
4. Assemble the temporal coding sequence by interleaving the Q harmonic subsequences according to the phase-shift rule in Equation (S9). This yields, for each meta-atom (n, m), a sequence of L digits representing one modulation period.
5. Export the resulting STC matrix as $N \times M$ rows and L columns. Each row contains the temporal coding sequence for one meta-atom and each entry belongs to the coding alphabet {0,1,2,3}.

Output: STC matrix used for the multifrequency synthesis.

Together with the objective functions and optimization parameters reported in the Experimental Section, this algorithmic summary provides the information needed to implement an independent version of the synthesis workflow and to reproduce the scattering calculations from the provided STC matrices.

STC Matrices

The STC matrices pertaining to the examples in Figure 3 and Figure 4 are provided as ASCII files. For identification, the data files are conventionally named "STCM_Figure_nf.txt", with *nf* denoting the figure number pertaining to the example. Thus, for instance, the data file named "STCM_Figure_3.txt" pertains to the example in Figure 3. Each data file is organized as a matrix of $N \times M$ rows and L columns containing the digits {0,1,2,3}. Accordingly, each row of the matrix contains the sequence of L digits that constitutes one period of the temporal coding for a given element. We follow a sequential arrangement along the y and z directions so that, for instance, the first row pertains to the element $(n = 1, m = 1)$, the second row to the element $(n = 2, m = 1)$, the $(N + 1)$th row to the element $(n = 1, m = 2)$, and so on.

Impact of Neglecting Curvature in the Syntheses

Figure S3 illustrates the consequences of neglecting curvature in the multifrequency synthesis. Specifically, for the same geometry and parameters considered in Figure 6, four anomalous reflections are synthesized toward $\varphi = 15°, 30°, -45°, -60°$, at the harmonic orders $\nu = 0, 1, 2, 3$, respectively. In this case, the synthesis is carried out under the assumption of a planar surface, using the analytical procedure in Ref. [S4], which yields the theoretical beam profiles shown as dashed curves. When the resulting STC matrices are instead applied to an actual curved geometry, such as the convex profile of Case A, the scattered responses differ substantially from the intended ones, as shown by the solid curves. These results clearly demonstrate that curvature effects, even for the mild conformal profiles considered in this study, play a critical role and cannot be neglected in the synthesis process.

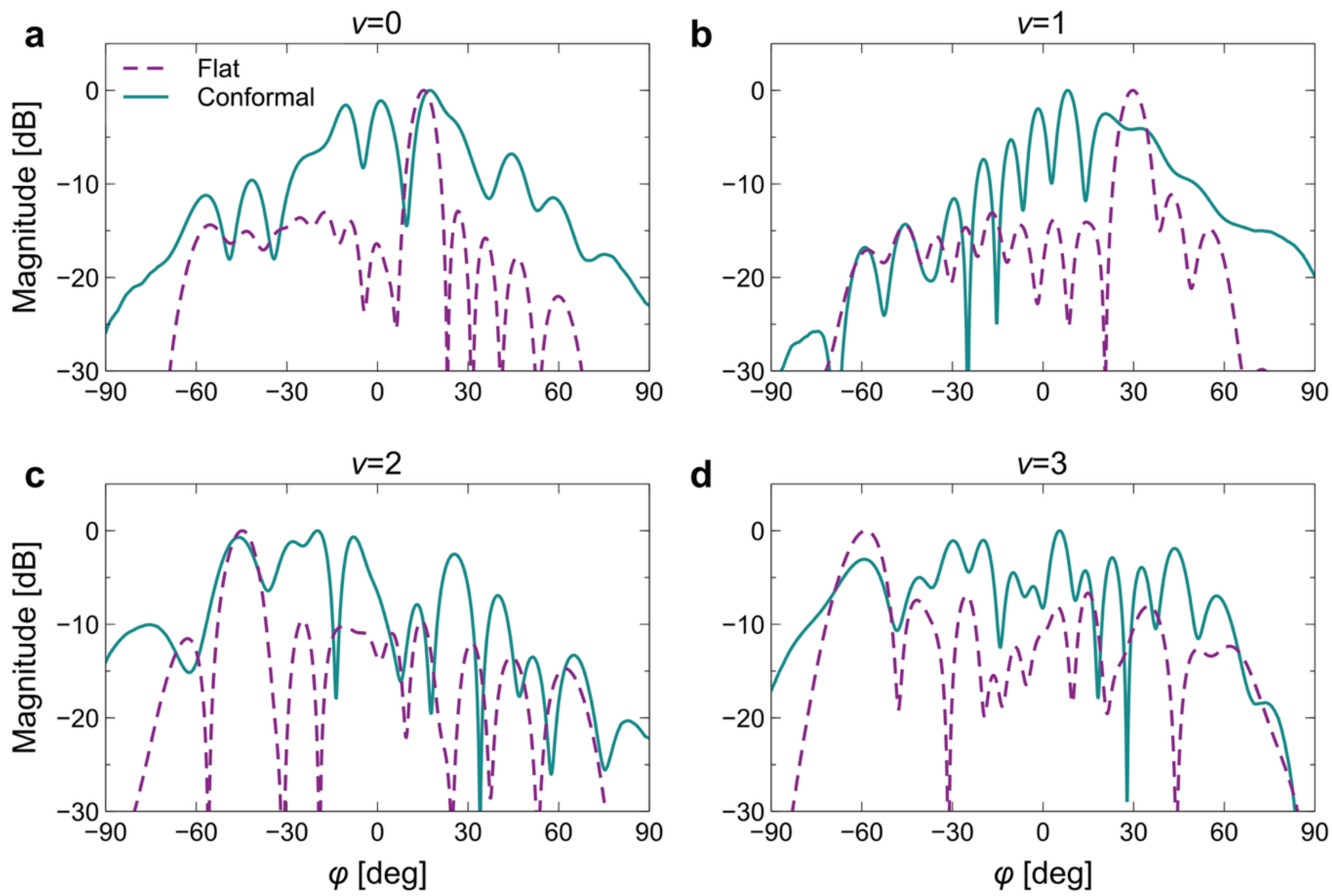

Figure S3 | Impact of neglecting curvature in the STC synthesis. a–d) Simulated azimuthal scattering patterns obtained at the harmonic orders $\nu = 0, 1, 2$, and 3, respectively, for fixed $\theta = 90°$, assuming axial invariance. The synthesized patterns correspond to anomalous reflections directed toward $\varphi = 15°$, $30°$, $-45°$, and $-60°$, respectively. Dashed curves correspond to patterns synthesized under the assumption of a planar metasurface, neglecting curvature effects. Solid curves show the resulting scattering responses when the same planar-designed STC sequences are applied to a conformal structure (Case A in Figure 2a, with $R = 300\text{mm}$ and $D = 224\text{mm}$). Results are obtained assuming a carrier frequency $f_c = 10.3$ GHz and $P = Q = S = 4$, corresponding to a sequence length $L = PQ = 16$. All patterns are normalized with respect to their maximum value.

References

S1. E.F. Knott, J.F. Schaeffer, M.T. Tulley, *Radar Cross Section* (Institution of Engineering and Technology, 2004).

S2. J. Baldauf, S.W. Lee, H. Ling, R. Chou, "On Physical Optics for Calculating Scattering from Coated Bodies," *Journal of Electromagnetic Waves and Applications* 3 (1989): 725–746, https://doi.org/10.1163/156939389X00403.

S3. W.R. Smythe, *Static And Dynamic Electricity* (Taylor & Francis, 1989).

S4. G. Castaldi, L. Zhang, M. Moccia, A.Y. Hathaway, W.X. Tang, T.J. Cui, V. Galdi, "Joint Multi-Frequency Beam Shaping and Steering via Space–Time-Coding Digital Metasurfaces," *Advanced Functional Materials* 31 (2021): 2007620, https://doi.org/10.1002/adfm.202007620.

S5. M.J.E. Golay, "Static Multislit Spectrometry and Its Application to the Panoramic Display of Infrared Spectra," *J. Opt. Soc. Am.* 41 (1951): 468–472.

S6. W. Rudin, "Some Theorems on Fourier Coefficients," *Proceedings of the American Mathematical Society* 10 (1959): 855–859.

S7. M. Moccia, S. Liu, R.Y. Wu, G. Castaldi, A. Andreone, T.J. Cui, V. Galdi, "Coding Metasurfaces for Diffuse Scattering: Scaling Laws, Bounds, and Suboptimal Design," *Advanced Optical Materials* 5 (2017): 1700455–11, https://doi.org/10.1002/adom.201700455.
